# Unveil Fundamental Graph Properties for Neural Architecture Search

**DOI:** 10.1002/advs.202516574

**Published:** 2026-02-23

**Authors:** Zhenhan Huang, Tejaswini Pedapati, Pin‐Yu Chen, Chunheng Jiang, Jianxi Gao

**Affiliations:** ^1^ Department of Computer Science Rensselaer Polytechnic Institute Troy USA; ^2^ IBM Research Yorktown Heights USA

**Keywords:** AI automation, deep learning, network property, neural architecture search

## Abstract

Deep learning profoundly impacts various areas, such as face recognition and language translation. Owing to the increasingly high computational costs of training neural architectures, it is intractable to manually examine the performance of various neural architectures, promoting the area of Neural architecture search (NAS) that enables artificial intelligence (AI) automation. Despite the advance of NAS in automatically discovering the optimal neural networks, the fundamental understanding of the structure of neural architectures is limited. To fill the gap, we propose a method dubbed *NASGraph* that converts neural architectures to graphs whose graph properties determine their performances, enabling us to search for or design strong architectures. On various standard NAS benchmarks, *NASGraph* outperforms baseline NAS methods and requires remarkably fewer computation resources. The numerical results illuminate the relationship between neural architecture and its performance, complementing other approaches. Therefore, combining our approach with these approaches leads to performance improvement, but not combining the others. Our study offers a new perspective on network science for AI, potentially advancing various aspects of machine learning and uncovering the black box of convolutional neural networks.

## Graphical abstract

This paper proposes NASGraph, a graph‐based framework that represents neural architectures as graphs whose structural properties determine performance. By revealing structure–performance relationships, NASGraph enables efficient neural architecture search with significantly reduced computation. Experiments on standard NAS benchmarks show superior performance and provide new network‐science insights into convolutional neural networks.

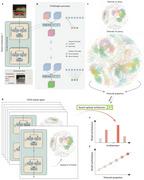

## 1 Introduction

Deep learning is reshaping modern society in many aspects, from identifying objects in images [1, 2, 3, 4, 5], to reasoning based on text prompts [6, 7, 8, 9, 10] and generating images for given text descriptions [11, 12, 13, 14, 15]. However, computational resource consumption is the biggest issue. On the one hand, training a neural network becomes computationally costly as the model size scales up. With a fixed model size, a larger training dataset generally leads to a better‐trained model. Hence, a trend exists to train a model from scratch, which consumes a large amount of computational resources. On the other hand, architectural design plays a crucial role. For example, adding residual connections [16] in the model can greatly alleviate gradient vanishing or exploding issues appearing in deep networks. Finding an excellent architecture, however, is non‐trivial: it requires specialized knowledge and a tremendous amount of computational time. Besides, the existing manually designed architecture inevitably introduces human bias, potentially ignoring architecture variants that may perform better.

Neural architecture search (NAS) aims to automate the process of discovering state‐of‐the‐art (SOTA) deep learning models. The objective of NAS is to find an optimal neural architecture:

(1) $$\min_{a\in A}\;F_{\mathrm{val}}\left(a,\theta^{*}\right)\;\text{s.t.}\;\theta^{*}={\text{argmin}}_{\theta }\;F_{\mathrm{train}}\left(a,\theta \right)\;.$$

Here F(a,θ) denotes the performance (e.g., a task‐specific loss function) of the neural architecture a trained for a fixed number of epochs using a dataset, and A is the search space. NAS techniques have shown competitive performance in various applications such as image classification [17, 18, 19, 20] and semantic segmentation [21, 22, 23, 24]. The pioneering work [25] based on reinforcement learning is resource intensive. To accelerate the search process, various approaches have been proposed, including weight sharing [26, 27], progressive complexity search stage [28, 29], gradient descent in the differentiable search space [30, 31, 32, 33, 34], predictor‐based NAS techniques [35, 36], Bayesian optimization [36, 37, 38], etc. The recent emergence of training‐free NAS [39, 40, 41] pushes the boundary of efficient NAS techniques further and greatly eases the computational burden. Training‐Free NAS uses a training dataset to compute a proxy metric in place of accuracy to rank the candidate architectures obtained by a single forward/backward propagation. It models trainability [42, 43, 44] and expressivity [45, 46, 47] to perform searches. However, despite finding some excellent architectures through search, the topological properties of these optimal neural architectures remain unknown, preventing us from fundamentally understanding neural network structures‐performance relationships. In NAS, the structure of the optimal neural architecture is searched and the corresponding optimal model weights are determined by the training dataset. Hence, we hypothesize that structure determines the performance of neural architectures.

Directly modifying the structure of neural architectures has been widely used to improve their performance. For example, in the structured network pruning, [48, 49, 50, 51], based on essential structural elements such as filters and layers. In the evolutionary NAS [52, 53, 54, 55, 56], model structures are constantly mutated to search for the optimal structure. Recently, two research directions have attempted to understand neural networks from a network science perspective by converting neural architectures to graphs. Relational graph [37, 57, 58, 59] considers connections between neural network layers or neural network blocks as graph edges and neural components as graph nodes that disregard the inherent difference between neural components. However, this approach oversimplifies the neural network model, and many configurations become indistinguishable. For another example, mapping a neural network to its line graph based on dynamical systems [60] also establishes the connection between the graph space and neural network space. Although this approach captures more details, the probing process requires training, which can be time‐consuming for large models. Nevertheless, the fundamental question *which topology makes an excellent neural network* remains unanswered.

## 2 Results

### 2.1 The Performance of the *NASGraph* Framework

**FIGURE 1:**
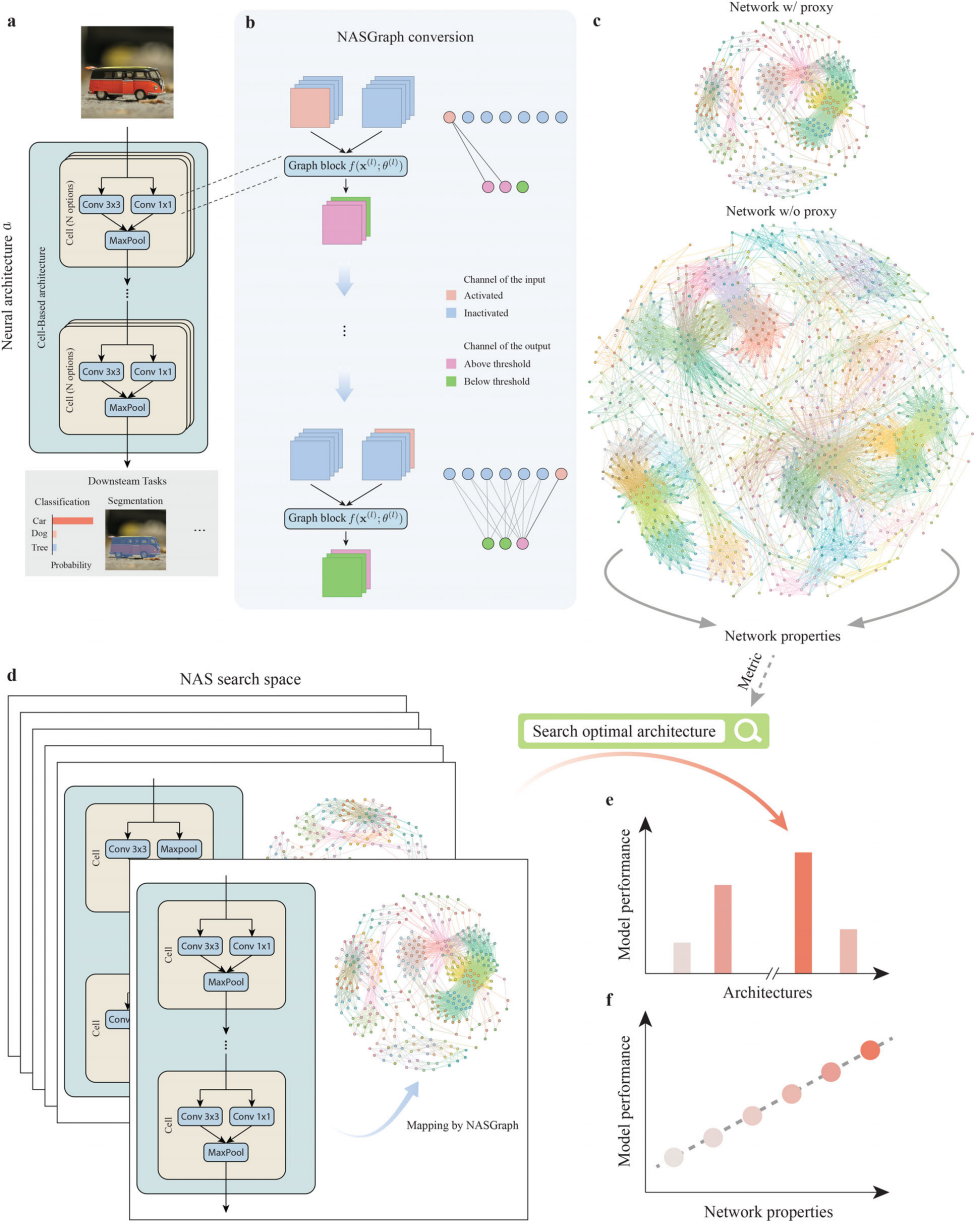
An overview of the *NASGraph* framework. (a) Input images are fed into neural architectures to obtain performance for various downstream tasks. Different options of cells comprise the search space A. The goal of the *NASGraph* framework is to find the neural architecture with the optimal performance. (b) Toy example of the conversion process to map a graph block to a subgraph. Conversions over all graph blocks constitute a graph. In each conversion step, only one channel of the input is activated. A forward propagation is performed to determine edge connections between nodes. (c) Graphs converted by the *NASGraph* framework. The proxy model is used to accelerate the search process. After graphs are established, neural architectures are searched based on graph properties. Owing to the high correlation between graph properties and the performance of neural architectures, no fitting is needed to predict, i.e., the search process is training‐free. (d) In the NAS search space, each neural architecture is uniquely mapped to a graph. (e) The goal of the *NASGraph* framework is to find the optimal neural architecture using graph properties as searching metrics. (f) Graph properties exhibiting correlation with the performance of neural architectures can be used as the training‐free NAS metric.

**FIGURE 2:**
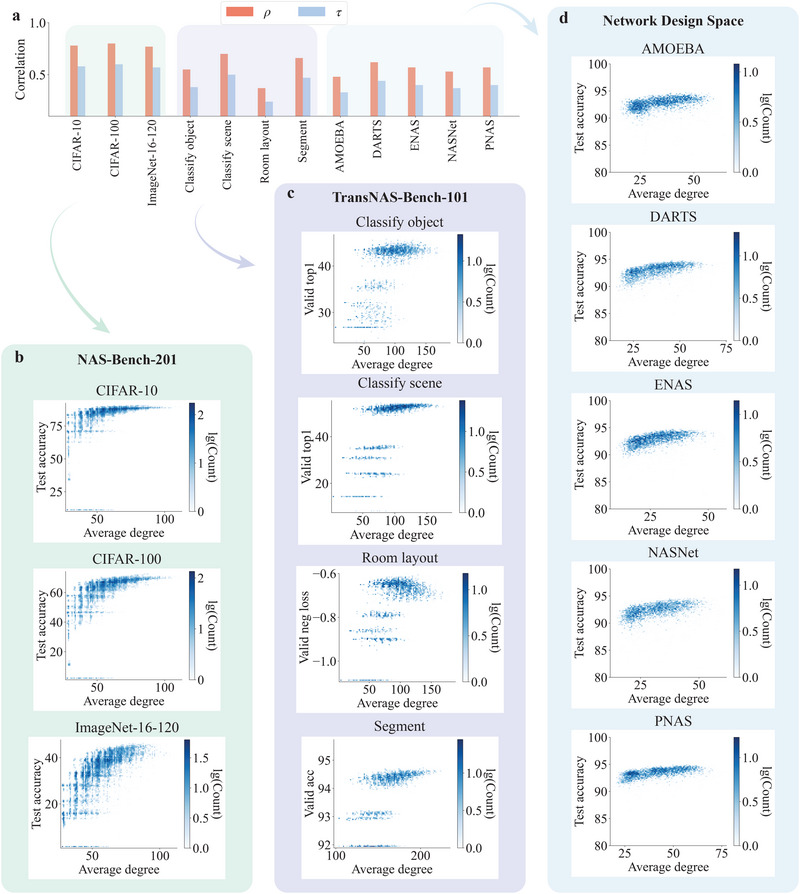
The ranking correlation between the performance of neural architecture a and the graph properties. (a) ranking correlation on different datasets for three benchmarks: (b) NAS‐Bench‐201, (c) TransNAS‐Bench‐101, (d) NDS. There are outliers (neural architectures with considerably lower test accuracy) in the NDS search space that are not shown in the visualization. They are considered in the computation of ranking correlation. In the *NASGraph* framework, each neural architecture is uniquely mapped to a graph, i.e., a↦G(V,E). Color map indicates the number of neural architectures with the same average degree and performance. The comparison of correlations with baseline methods is reported in Tables S1–S3, Supporting Information.

We address these challenges by developing a *NASGraph* framework that maps a neural network to a graph whose property reflects the neural network performance, as shown in Figure 1f. Specifically, we define the inputs of each layer in the neural network as nodes in the graph. If a non‐zero input in the first layer results in a non‐zero input to the adjacent layer, we create a link between them (See **Methods** section for details). Once the graph is created, we extract the associated graph properties as NAS performance metrics to rank neural architectures on extensive standard NAS benchmarks. Figure 2a shows the Spearman's ranking correlation ρ and Kendall's Tau correlation τ between model performances and graph properties on various standard NAS benchmarks. Figure 2b–d shows the correlation between test accuracy and average degree, where color indicates the number of architectures with the same performance and graph property. On all benchmarks, the average degree positively correlates with its performance, and many other graph properties (e.g., density, resilience, and wedge) strongly correlate with the neural architecture performance, reported in Supporting Information S.1.1, and Figures S3 and S4 (Supporting Information). The consistency in the correlation over a wide range of benchmarks exhibits the effectiveness of the *NASGraph* framework.

To better understand the topological properties of cell‐based NAS benchmark (such as The NAS‐Bench‐201 [61]), we compare the best and worst neural architectures found by the *NASGraph* framework as shown in Figure 5. The neural architecture with superior performance is converted to a remarkably denser graph than the one with inferior performance. The architecture with the highest score selected by avg_deg is the same as that chosen by the training‐free NAS metric synflow [62]. The advantage of our approach is that it offers an initiative understanding of the reason for excellent performance, which is different from the synflow based on the lottery ticket hypothesis [63], although both methods are data‐agnostic.

**FIGURE 3:**
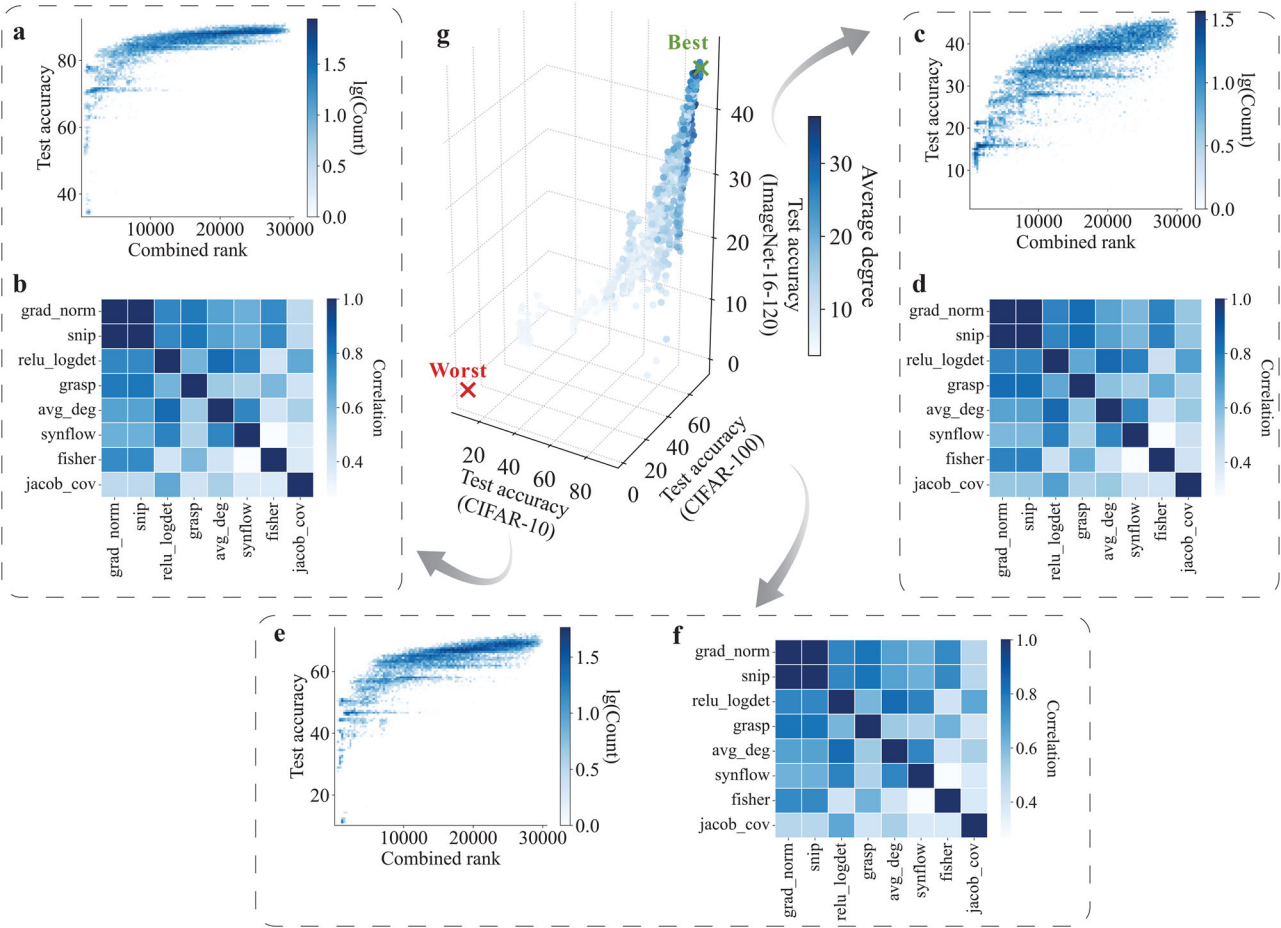
Correlation between test accuracy and combined rank (rank(avg_deg) + rank(jacob_cov)) of neural architectures. Correlation in the NAS‐Bench‐201 search space on various datasets: (a) CIFAR‐10. (c) CIFAR‐100. (e) ImageNet‐16‐120. The correlation between training‐free NAS methods on different datasets: (b) CIFAR‐10. (d) CIFAR‐100. (f) ImageNet‐16‐120. (g) Visualization of the neural architectures on three different datasets. Neural networks with a high average degree perform well across different datasets, validating the effectiveness of the data agnostic property of the *NASGraph* method.

**FIGURE 4:**
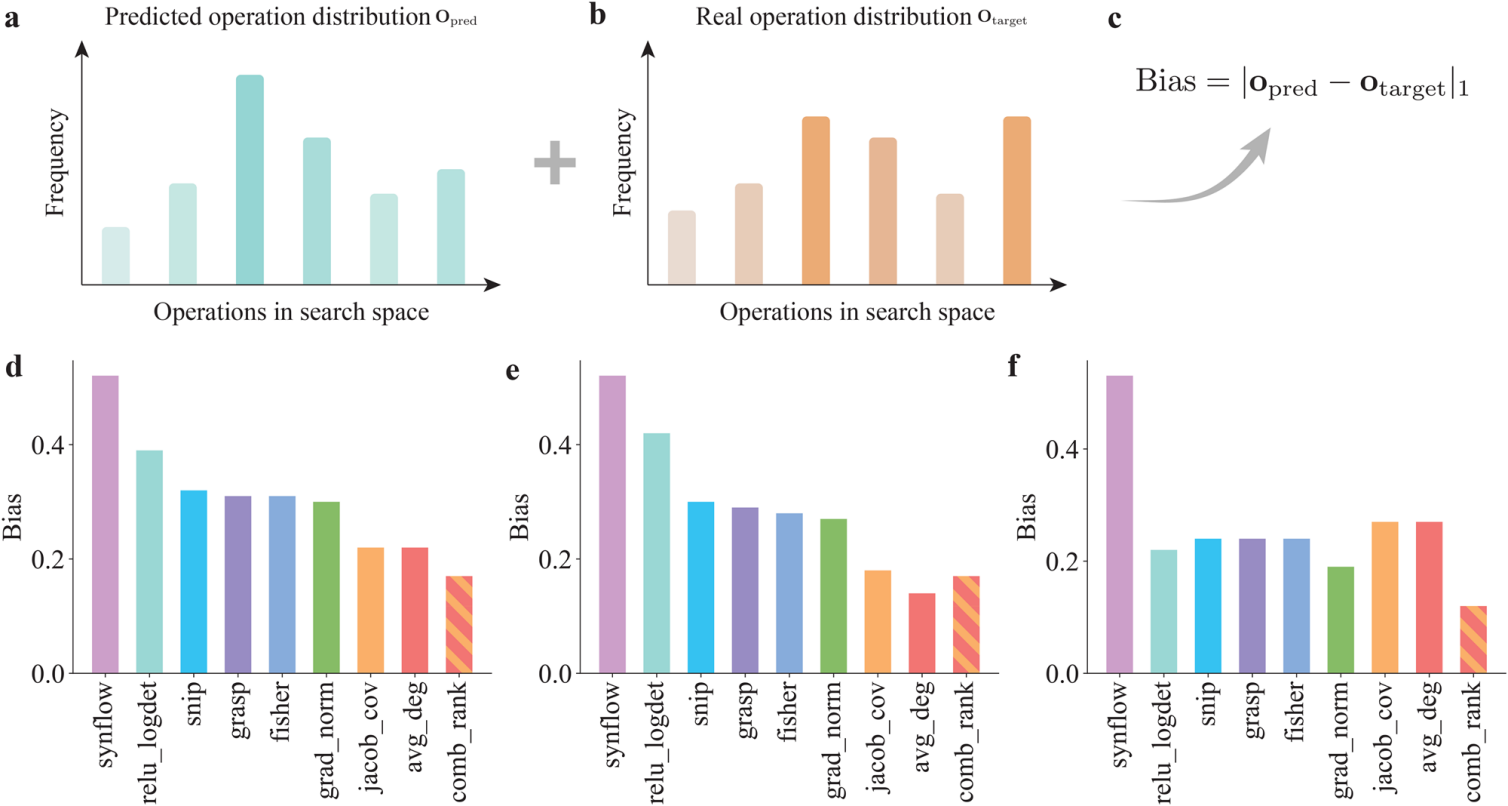
Comparison of the bias for training‐free NAS methods. Bias is computed based on (a) the predicted operation distribution $O_{\mathrm{pred}}$ and (b) the real operation distribution $O_{\mathrm{target}}$. Both (a) and (b) are schematic illustrations of the frequency distribution of operations. (c) The bias calculation is based on the $l_{1}$‐norm between these two distributions. The bias for neural architectures in the NAS‐Bench‐201 search space trained on various datasets is compared: (d) CIFAR‐10. (e) CIFAR‐100. (f) ImageNet‐16‐120.

**FIGURE 5:**
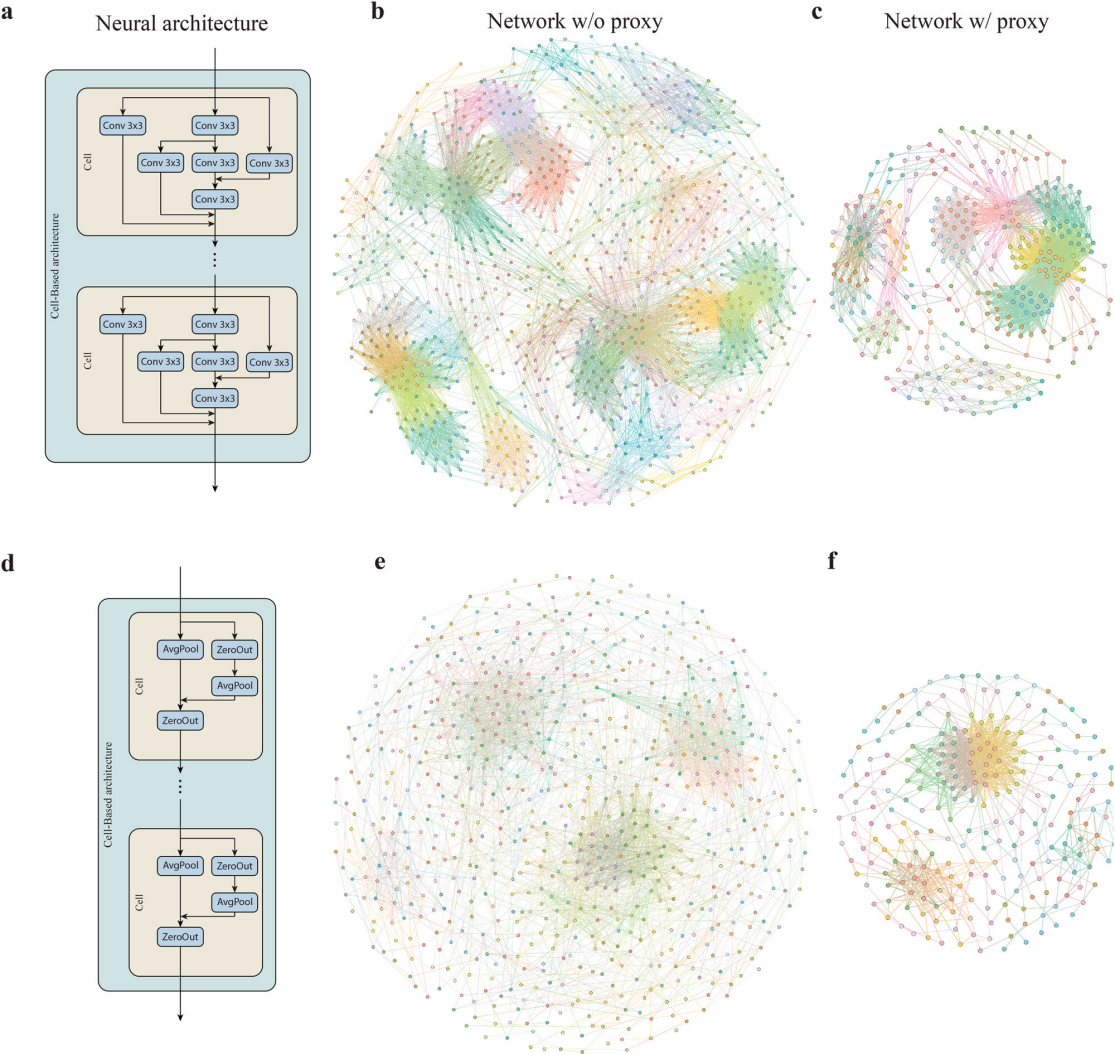
Visualization of the best and worst neural architectures in the NAS‐Bench‐201 search space. Given a neural architecture a, it is uniquely mapped to a graph G(V,E). The proxy model is used to reduce the search cost for discovering neural architectures. (a) The neural architectures with the highest test accuracy. (b) Graph corresponding to the best neural architecture without using the proxy settings. (c) Graph corresponding to the best neural architecture using the proxy setting. (d) The neural architectures with the lowest test accuracy. (e) Graph corresponding to the worst neural architecture without using the proxy settings. (f) Graph corresponding to the worst neural architecture using the proxy setting.

### 2.2 Comparison with Baseline Approaches

To further demonstrate the effectiveness of our *NASGraph* framework, we compare it with other baseline methods from three aspects: datasets, search spaces, and cross‐tasks. First, using NAS‐Bench‐201 [61], we compare the ranking correlation between *NASGraph* and other training‐free NAS methods on three datasets shown in Supporting Information Table S1. Our method outperforms the baseline methods with excellent correlations, reliability for various real‐world datasets, and robustness for random initialization of model weights. Details about metric variation over different runs are reported in Supporting Information S.1.4. Second, for the same dataset, we demonstrate that our method performs superior in the five search spaces of NDS benchmark [64], such as AMOEBA, DARTS, ENAS, NASNet, and PNAS. The results are shown in Supporting Information Table S2. Third, to examine the performance of the proposed method in cross‐task NAS, we apply the *NASGraph* framework in TransNAS‐Bench‐101 [65], as shown in Supporting Information Table S3. We notice that jacob_cov performs best in this benchmark but is relatively inferior on other benchmarks, a common issue for NSA metrics [66, 67]. The performance of different graph properties is included in Supporting Information Table S11. In addition to comparing ranking correlation, we examine the performance of searched neural architectures within a fixed search space on Imagenet under the mobile setting [68, 69] (See Method). The performance comparison in Supporting Information Table S6 demonstrates that our approach performs better than baseline NAS methods. Overall, our method demonstrates excellent performance across all tasks.

### 2.3 Graph Property of *NASGraph* Complements Existing NAS Metrics

The *NASGraph* framework not only demonstrates excellent performance but also provides a unique perspective for understanding artificial neural networks through the lens of network science. Our approach is not merely a simple modification. While it is common to combine different techniques to enhance model performance, significant improvements are often observed when the combined approaches are quite different from each other. Therefore, we investigate the performance of integrating *NASGraph* framework with baseline metrics. Specifically, two metrics are combined by summing rankings of neural architectures by two metrics. We use a combination of avg_deg and jacob_cov metrics to predict model performance, i.e., rank(avg_deg) + rank(jacob_cov). For the case of tied ranking, we use the average values. By combining avg_deg with jacob_cov, τ increases from 0.58 to 0.66 on CIFAR‐10 (14% improvement), from 0.60 to 0.67 on CIFAR‐100 (12% improvement), from 0.57 to 0.62 on ImageNet‐16‐120 (12% improvement). The combined metric can outperform all existing combinations reported in [40, 69], as shown in Supporting Information Table S5. Figure 3a,c,e shows the correlation between combined metric and model performance. More importantly, our findings reveal an intriguing consistency: despite the significant variations in accuracy across different datasets, the *NASGraph* method reliably ranks neural architectures irrespective of the training dataset used. As illustrated in Figure 3g, the performance of these architectures on three distinct datasets showcases a remarkable data‐agnostic property. This evidence strongly suggests that the top‐performing architectures are often associated with denser graphs, underscoring the potential advantages of employing our method in neural architecture evaluation.

We compute the ranking correlation between each pair of training‐free NAS metrics. When ρ=1, two metrics provide an identical rank. Figure 3b,d,f shows the pairwise correlation on three datasets: CIFAR‐10, CIFAR‐100 and ImageNet‐16‐120. The correlation between avg_deg and jacob_cov is low (0.55), but the combination of these two metrics gives the optimal performance, indicating a complementarity between avg_deg and jacob_cov. Note that grad_norm and snip do not give the same rankings of all neural architectures. When we check the architecture rankings precisely using these two metrics, we find ρ=0.9982 on CIFAR‐10, ρ=0.9984 on CIFAR‐100, and ρ=0.9988 on ImageNet‐16‐120. Our results show that the graph property of *NASGraph* complements existing NAS metrics.

### 2.4 The Bias to NAS Operations

Next, we examine the reasons behind the excellent performance of *NASGraph* and its integration with baseline approaches from the bias perspective. Increasing evidence suggests that many NAS metrics inherently favor certain operations while neglecting others within a search cell. This phenomenon is known as operation bias [66, 67], and it can lead to performance degradation.

To investigate this bias, we extract the top 10% of neural architectures from the NAS‐Bench‐201 benchmark and count the frequency of each NAS operation (avg_pool, none, nor_conv_1×1, nor_conv_3×3, skip_connect) in the selected subset. We illustrate the predicted distribution of operations in Figure 4a and the real distribution in Figure 4b. The frequency difference between predicted operation distribution and real distribution constitutes the bias toward operations in the NAS search space:

(2) $$\text{Bias}=|O_{\mathrm{pred}}-O_{\mathrm{target}}{|}_{1}\;,$$

where O is the distribution of operations, i.e., frequencies of operations in the search space, as shown in Figure 4c. We show the comparison of bias for training‐free NAS methods in Figure 4d–f. Compared to baseline methods, our method shows a low bias. Overall, combining avg_deg with jacob_cov gives the best prediction on the operation distribution. Averaged bias over three datasets is reported in Supporting Information Table S4.

The predicted operation distribution, in contrast to the real distribution, is shown in Supporting Information Figure S2. Our *NASGraph* framework shows a relatively low preference for skip_connect, whereas jacob_cov demonstrates a strong preference for it. As a result, integrating *NASGraph* with jacob_cov leads to a more balanced selection of skip_connect, partially explaining why this combination performs the best among all combined metrics. Another contributing factor relates to the properties of jacob_cov. The Jacobian for the i‐th neuron in the output of the layer l with parameter $\theta_{\alpha }$ evaluated at a point **x** is defined as [39] $J_{i\alpha }\left(x^{\left(l\right)}\right)=\partial_{\theta_{\alpha }}z_{i}^{\left(l\right)}\left(x^{\left(l\right)}\right)$. While the jacob_cov metric considers the gradient of model parameters and focuses on the backpropagation process, the *NASGraph* framework considers the forward propagation for each graph block. Consequently, they complement each other, and the corresponding operation distribution is close to GT, resulting in a higher ranking correlation, as shown in Supporting Information Figure S2a–c.

### 2.5 Efficiency Analysis

**FIGURE 6:**
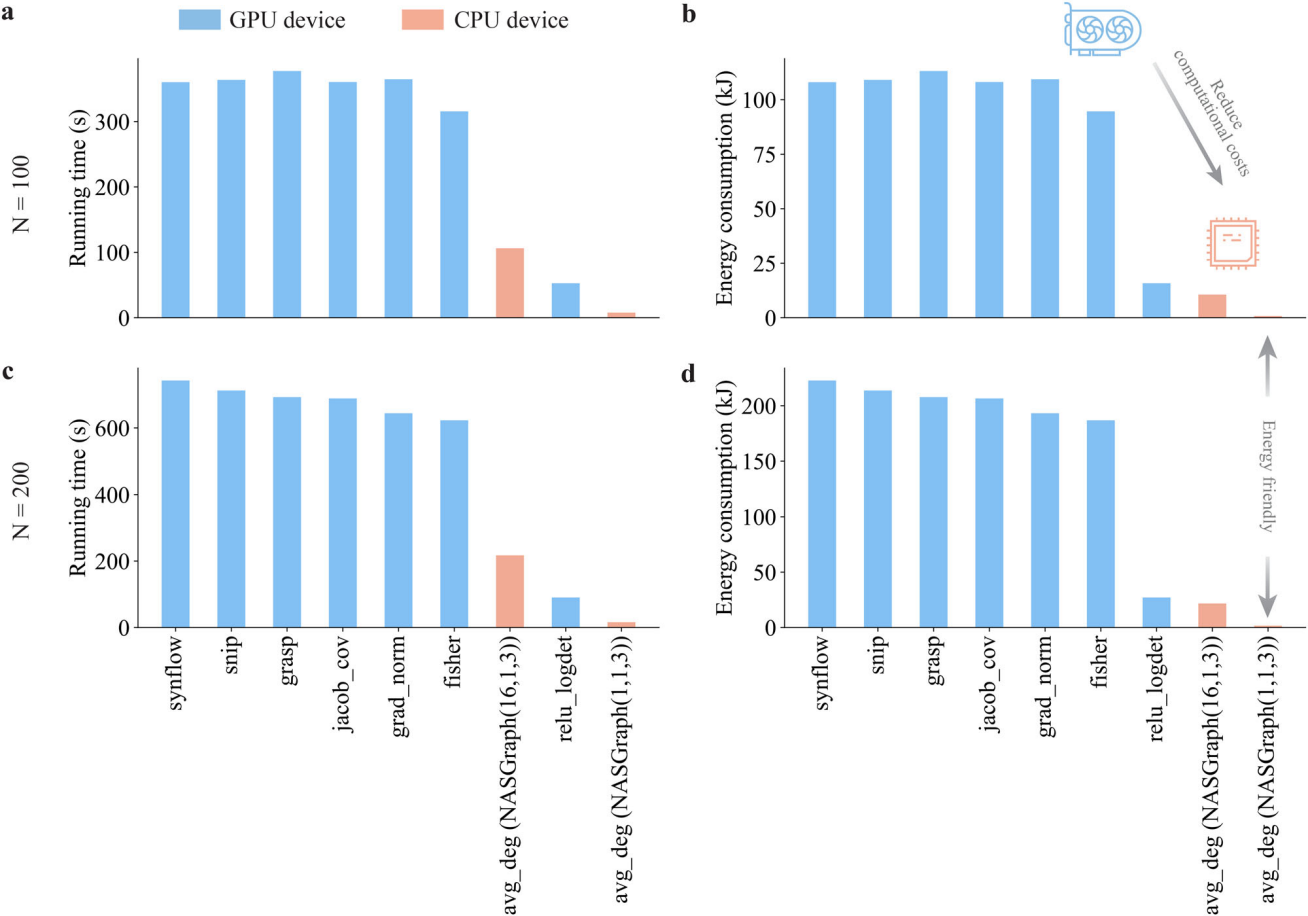
Comparison of the efficiency using the random search method. Architectures in the NAS‐Bench‐201 benchmark are searched to find the optimal ones. Different number of architectures N is sampled from the search space for comparing the search efficiency: (a) N=100, (b) N=200. (c) We estimate the energy consumption using the multiplication of running time (s) and energy consumption rate (W) for two random search settings: (c) N=100, (d) N=200.

In addition to performance, computational efficiency is essential for excellent algorithms. To demonstrate the efficiency of our *NASGraph* framework, we compare the running time and the mean and standard deviation of test accuracy with the highest training‐free metrics in Supporting Information Table S7. The “GT” in the table represents the highest test accuracy among the N sampled neural architectures, showing that our approach not only performs comparably to the GT but also requires less time. It is important to note that while all baseline methods rely on GPU time, our method only utilizes CPU time. Despite the CPU and GPU differences, our method is faster than all baseline methods except for relu_logdet. Although it sacrifices some accuracy, the more lightweight surrogate model, NASGraph(1, 1, 3), achieves the fastest efficiency, requiring only 15% and 17% of the time needed by the fastest baseline method for a random search. Figure 6 shows the comparison of running time in the random search and that of energy consumption. The *NASGraph* framework is lightweight and remarkably reduces the computational costs.

Next, we illustrate that the efficiency of our *NASGraph* framework stems from its two effective components: the first converts neural architectures into graphs, and the second computes the graph properties. The graph conversion process involves a single forward propagation without backpropagation to compute gradients, which enhances efficiency. The computational overhead associated with calculating graph properties is generally minimal. For example, given a graph G(V,E) where $\left|V\right|=n_{g}$ and $\left|E\right|=m_{g}$, the time complexity for computing the average degree is $O(m_{g}+n_{g})$. The computational time for other graph properties is detailed in Supporting Information Table S8.

## 3 Discussion

A fundamental question in deep neural networks is understanding the relationship between their structural configuration and performance. Increasing evidence indicates that manually designed neural networks exhibit repeated structural patterns. For example, residual connections [16] are widely utilized in CNNs [70, 71, 72] and transformers to enhance their performance [73]. Neural Architecture Search (NAS) aims to discover better neural architectures iteratively, but it falls short of uncovering the fundamental principles that govern the relationship between structure and performance. Despite recent efforts to map neural networks to relational graphs [57] or model the training processes as networked dynamical systems [60], we still cannot answer these fundamental questions. Inspired by the understanding that the forward propagation response from the input to its output contains valuable information about the original neural networks, we have developed a framework called *NASGraph* to provide insights into the architectural design of neural networks.

Our work introduces a novel mapping strategy that converts neural networks into graph representations. Each node in the graph corresponds to the inputs or outputs of the layers, and the connections between them denote the forward propagation process, depicting the pathways through which data flows. This method allows us to uniquely represent neural networks as graphs G(V,E). Our empirical analysis shows that specific properties of graphs, such as average degree, serve as effective indicators of a neural network's performance. We discovered that they frequently outperform traditional NAS metrics by systematically examining these properties against established Neural Architecture Search (NAS) benchmarks. Additionally, the *NASGraph* framework is notable for its high efficiency in computing graph properties, making it an exceptionally effective tool for searching and refining neural networks and ultimately leading to improved performance across various applications. Our findings not only highlight the potential of graph properties as performance indicators but also establish a powerful and efficient means of optimizing neural networks for future advancements in the field. In the context of neural architecture design, the *NASGraph* framework facilitates the comparison of candidate models without requiring training, enabling efficient evaluation by eliminating the computational cost associated with model training.

We intend to explore several significant avenues moving forward. First, we will extend our efforts to benchmark against standard NAS metrics specifically for transformer architectures, ensuring our methods remain both applicable and competitive. Second, we plan to investigate innovative strategies for modeling graph nodes while balancing the tradeoff between efficiency and complexity. Finally, we are dedicated to designing an efficient algorithm and developing pioneering neural network models to guide us toward optimal performance.

## 4 Materials and Methods

### 4.1 Converting Neural Architecture to Graph

We propose a method dubbed *NASGraph* to convert the neural architectures to graphs. The *NASGraph* framework is shown in Figure 1. A neural architecture is uniquely mapped to a graph, i.e., a↦G(V,E).

Figure 1a shows the neural architecture a. All possible cells comprise the search space A. The basic element in the *NASGraph* framework is graph block. An example of a graph block is Conv‐BN‐ReLU (convolution layer followed by batch normalization layer, and then ReLU activation function). We use the notation $f^{\left(l\right)}\left(x^{\left(l\right)};\theta^{\left(l\right)}\right)$ to represent the l‐th graph block, where $x^{\left(l\right)}$ is the input to the graph block and $\theta^{\left(l\right)}$ is the model parameter. Figure 1c shows the converted graph G(V,E). To accelerate the search process, we use the proxy model to reduce the model complexity. Graph nodes correspond to inputs to graph blocks, and graph edges are determined by the forward propagation over graph blocks.

We convert each graph block independently. All‐ones matrix is used as the input to the graph block in the forward propagation process. For the l‐th graph block, the input is $x^{\left(l\right)}=1^{C^{\left(l-1\right)}\times H^{\left(l-1\right)}\times W^{\left(l-1\right)}}$, where $C^{\left(l-1\right)}$ is the number of channels, $H^{\left(l-1\right)}$ is the image height, and $W^{\left(l-1\right)}$ is the image width. All‐ones matrix provides an unbiased estimate of the contribution of the input to the output. The contribution is determined by the graph block itself. Further, to determine the contribution of the c‐th channel of the input on every channel of the output for the l‐th graph block, we apply a mask $M_{c}^{\left(l\right)}$ to the input so that only the c‐th channel ${\left(x_{d_{1}d_{2}d_{3}}^{\left(l\right)}\right)}_{d_{1}=c}$ is an all‐ones matrix $1^{H^{\left(l-1\right)}\times W^{\left(l-1\right)}}$ and other channels are zero matrices $O^{H^{\left(l-1\right)}\times W^{\left(l-1\right)}}$. We evaluate the contribution of the c‐th channel ${\left(x_{d_{1}d_{2}d_{3}}^{\left(l\right)}\right)}_{d_{1}=c}$ to the output $y^{\left(l\right)}$ by performing a forward propagation as described by:

(3) $$y_{c}^{\left(l\right)}=f^{\left(l\right)}\left(M_{c}^{\left(l\right)}⊙x^{\left(l\right)};\theta^{\left(l\right)}\right)\;,$$

where $f^{\left(l\right)}\left(\cdot \right)$ is the l‐th graph block, ⊙ is the Hadamard product, and $\theta^{\left(l\right)}$ are the parameters of the l‐th graph block. The score $\omega_{i^{\left(l-1\right)}j^{\left(l\right)}}$ for the edge $e_{ij}$ between the graph node $i^{\left(l-1\right)}$ and $j^{\left(l\right)}$ is determined by:

(4) $$\omega_{i^{\left(l-1\right)}j^{\left(l\right)}}=\sum_{d_{2}=1}^{H^{\left(l\right)}}\sum_{d_{3}=1}^{W^{\left(l\right)}}{\left({\left(y_{i}^{\left(l\right)}\right)}_{d_{1}d_{2}d_{3}}\right)}_{d_{1}=j}\;.$$

If $\omega_{i^{\left(l-1\right)}j^{\left(l\right)}}$ is larger than 0, we build an edge between node $i^{\left(l-1\right)}$ and node $j^{\left(l\right)}$ that indicates the connection between i‐th channel of the input $x^{\left(l\right)}$ and j‐th channel of the output $y^{\left(l\right)}$. Otherwise, there is no connection. We use a virtual input graph block of identity operation to take the input to the neural architecture into consideration. After iterating over all graph blocks, we can uniquely construct a graph G(V,E).

When there are outputs from multiple graph blocks combined as the input to the same graph block. There are two ways of combining them. One is the concatenation while the other is the summation. These two ways are expressed by:

(5) $$\begin{array}{cc} & \text{Summation}=\sum_{\ell =l-k}^{l-1}x^{\left(\ell \right)}\in R^{C\times H\times W}\;, \end{array}$$

(6) $$\begin{array}{cc} & \text{Concatenation}=\left[x^{\left(l-1\right)},\text{…},x^{\left(l-k\right)}\right]\in R^{(C^{\left(l-1\right)}+\cdots +C^{\left(l-k\right)})\times H\times W}\;. \end{array}$$

Our proposed method is able to incorporate these two cases. Details regarding dealing with these two cases are shown in Supporting Information Section S.1.3.

Figure 1b shows a toy example. There are two inputs to the graph block and both inputs have 4 channels. The conversion process for this graph block has 8 steps. The first step has the first channel of the left input to be all‐ones matrix 1 while the rest of the channels be all‐zeros matrix O. The forward propagation of the first step determines the connection between the first channel of the input mapped to the red and blue graph nodes and all channels of the output mapped to the pink and green graph nodes. In the last step, a subgraph is built. The conversion process in the *NASGraph* framework is shown in the Algorithm 1. We use a threshold $m_{th}=0$ to determine whether an edge is built.

ALGORITHM 1 **Converting Neural Architecture to Graph in *NASGraph* Framework**

**Table:** 

1:	**Input**: A neural architecture with Gaussian initialization, a=f(x|θ), $\theta \overset{i.i.d.}{∼}N\left(\mu ,\sigma^{2}\right)$ and a threshold $m_{th}$
2:	**for** l=1:ℓ **do** ▹ Loop through ℓ graph blocks
3:	**for** c=1:C **do** ▹ This step can be done in parallel, see SI S.1.2
4:	Applying the forward propagation according to Equation 3
5:	Compute $\omega_{i^{\left(l-1\right)}j^{\left(l\right)}}$ according to Equation 4
6:	**if** $\omega_{i^{\left(l-1\right)}j^{\left(l\right)}}>m_{th}$ **then**
7:	Build an edge between the graph node $v_{i^{\left(l-1\right)}}$ and $v_{j^{\left(l\right)}}$
8:	**end if**
9:	**end for**
10:	**end for**
11:	**Output**: A graph G(V,E)

We compare the correlation between graph properties and neural architecture performance with training‐free NAS proxies. zico [41] based on the theory of Gram Matrix [45] relates the training convergence rate and generalization capacity to mean and standard deviation of the gradients. relu_logdet (also dubbed naswot) [39] applies the theory on the number of linear regions to represent the model expressivity. jacob_cov [39] is based on the correlation of Jacobians with inputs. The model performance is negatively correlated to the correlation as the model can differentiate different inputs well [40]. grad_norm [40] sums the Euclidean norm of the gradients. It is consistent with the traditional network pruning theory: a larger magnitude of gradients indicates the importance of the model parameters. snip [74] is, based on the saliency metric in the network pruning [74], related to the connection sensitivity of neural network model. grasp [75] is based on the assumption that gradient flow is preserved in the efficient training. fisher [76] estimates fisher information of model parameters, synflow [62] preserves the total flow of synaptic strength.

### 4.2 Surrogate Model to Improve the Efficiency

To reduce computational overhead, NAS typically uses a training‐reduced proxy to obtain the performance of neural architectures. A systematic study is reported in EcoNAS [77] where four reducing factors are analyzed: (1) number of epochs, (2) resolution of input images, (3) number of training samples, (4) number of channels for Convolution Neural Networks (CNNs). To accelerate *NASGraph* framework, we also consider the surrogate models, i.e., models with computationally reduced settings. We dub the surrogate model *NASGraph*(c, n, m), where c is the number of channels, e is the number of search cells in a module, and m is the number of modules. The number of channels c corresponds to the output channel of the steam layer of the model. Taking architectures on the NAS‐Bench‐201 benchmark as an example, the stem layer maps input $f_{\mathrm{stem}}:R^{3\times H\times W}\to R^{C\times H^{\prime }\times W^{\prime }}$. The dimension C is the number of channels for the surrogate model *NASGraph*(c, n, m). We use the surrogate model *NASGraph*(16, 1, 3) as the default model in the *NASGraph* framework.

### 4.3 Search Neural Architectures in a Fixed Search Space

Given a fixed search space A, we use avg_deg as the metric to predict the performance. We use DARTS [30] search space to sample neural architecture candidates and the ImageNet dataset [78] to train and evaluate model performance. Following the setting in [32, 68, 69], neural architectures are stacked with 14 cells and the initial channel number is set to be 48. The spatial resolution is downscaled to 28×28 given the input image size of 224×224 using the first three convolution layers of stride 2. The performance of neural architectures with the highest avg_deg score is used to report the performance of the searched neural architecture.

### 4.4 Random Search

We evaluate the effectiveness and efficiency of the *NASGraph* framework using the random search algorithm as shown in Algorithm 2. A total number of N neural architectures are randomly sampled from the same benchmark. Metrics are computed as scores by a single forward or backward propagation over neural architectures with randomly initialized parameters. Scores are used to rank neural architectures. The performance of the neural architecture with the highest score is reported as the best performance of sampled N architectures. We test the proposed framework on the NAS‐Bench‐201 benchmark. N=100 and N=200 neural architectures are randomly sampled. Randomly initialized model weights follow a Gaussian distribution. The random search process is repeated 100 times. We report the mean and standard deviation of the test accuracies associated with the highest scores.

ALGORITHM 2 **Random Search Algorithm Using Single Metric**

**Table:** 

1:	net_generator = RandomGenerator()
2:	score_highest, net_best = None, 0
3:	**for** i=1:N **do**
4:	net = net_generator.pick_net()
5:	score = ComputeMetric(net)
6:	**if** score > score_highest **then**
7:	score_highest = score
8:	net_best = net
9:	**end if**
10:	**end for**
11:	acc_best = ExtractAccFromBenchmark(net_best)

### 4.5 Graph Property Computation

After converting neural architectures to graphs G(V,E) ($\left|V\right|=n_{g}$ and $\left|E\right|=m_{g}$) using *NASGraph*, we compute graph properties as NAS proxies. Four graph properties of average degree, density, resilience parameter [79], and wedge count [80] are examined for searching neural architectures. ① The **average degree** $\bar{k}$ calculates the average number of edges for one graph node. The average degree is expressed by $\bar{k}=\frac{1}{n_{g}}\sum_{i\in V}k_{i}$, where $k_{i}$ is the degree of node i. ② The **density** $d_{G}$ measures the ratio of the total number of edges to the maximum number of possible edges, $d_{G}=\frac{m_{g}}{n_{g}\left(n_{g}-1\right)}$. ③ The **resilience parameter** $\beta_{\mathrm{eff}}$ of a DAG [79] is defined by $\beta_{\mathrm{eff}}=\frac{1^{T}As^{\mathrm{in}}}{1^{T}A1}=\frac{\left\langle s^{\mathrm{out}}s^{\mathrm{in}}\right\rangle }{\left\langle s\right\rangle }$, where $1={\left(1,\text{…},1\right)}^{T}$ is the all‐ones vector, $s^{\mathrm{in}}=\left(s_{1}^{\mathrm{in}},\text{…},s_{n}^{\mathrm{in}}\right)$ is the vector of incoming degrees, and A is the adjacency matrix of the graph. ④ The **wedge count** $W_{G}$ counts the number of wedges [80], and a wedge is defined as a two‐hop path in an undirected graph. It is related to the triangle density of an undirected graph. The wedge count is expressed by $W_{G}=\sum_{i\in V}\frac{1}{2}k_{i}\left(k_{i}-1\right)$. Supporting Information Table S8 summarizes these graph properties and their computation complexity.

## Conflicts of Interest

The authors declare no conflict of interest.

## Supporting information


**Supporting File**: advs74506‐sup‐0001‐SuppMat.pdf

## Data Availability

The data that support the findings of this study are available from the corresponding author upon reasonable request.
