# Supplementary material for: Unveil Fundamental Graph Properties for Neural Architecture Search
Source: Adv Sci (Weinh). 2026 Feb 23;13(25):e16574. doi: 10.1002/advs.202516574 (PMC13137813; doi:10.1002/advs.202516574)
Supplement: Supplementary file 1 — Supporting File: advs74506‐sup‐0001‐SuppMat.pdf [file ADVS-13-e16574-s001.pdf]

S.1.1 Performance of Other Graph Properties

In addition to the average degree (`avg_deg`), we examine the performance of density (`density`), resilience parameter (`resilience`), and wedge count (`wedge`) as the training-free NAS proxy to rank neural architectures. Figure S3 shows the correlation on the NAS-Bench-201 benchmark. Graph properties exhibit a similar performance. The performance on the TransNAS-Bench-101 is shown in Figure S4. The performance difference among graph properties is significant. Besides, we notice that graph properties do not behave consistently on this benchmark. For the class object task, resilience has the best performance. For the segment semantic task, wedge becomes the best graph property.

S.1.2 Experiment Setup

Implementation details The establishment of *NASGraph* is block-wise. For each block, we "activate" (by setting all-ones matrix $(\mathbf{x})_{d_1 d_2 d_3} = \mathbb{1}^{H\times W}, 1 \leq d_1 \leq C$) channels of input data one by one. Since the computation for channels is independent, we apply the trick of parallel computing. We concatenate inputs for the same graph block on the batch dimension $[\mathcal{M}_1 \odot \mathbf{x}, \ldots, \mathcal{M}_c \odot \mathbf{x}]$ so that scores can be computed independently and in parallel. Under this condition, the batch size is equal to the input channel size. Using this approach, the batch size dimension has a different meaning than the standard definition in batch normalization. Moreover, the effective batch dimension is essentially one as we only use the same input $\mathbf{x}$ to determine scores. Therefore, we remove batch normalization during graph conversion in the entire neural architecture. For example, Conv-BN-ReLU becomes Conv-ReLU.
NAS benchmarks To examine the effectiveness of our proposed *NASGraph*, we use neural architectures on NAS-Bench-201 [61], TransNAS-Bench-101 [65] and Network Design Space (NDS) [64]. NAS-Bench-201 is built for prototyping NAS algorithms. It contains 15,625 neural architectures trained on CIFAR-10 [81], CIFAR-100 [81] and ImageNet-16-120 [82] datasets. The training statistics are reported at the 200th epoch for these three datasets. In addition to standard NAS benchmarks, we also examine the performance of *NASGraph* on TransNAS-Bench-101, specifically the micro search space. The micro (cell-level) TransNAS-Bench-101 has 4,096 architectures trained on different tasks using the Taskonomy dataset [83]. The NDS benchmark includes AmoebaNet [18], DARTS [30], ENAS [84], NASNet [25] and PNAS search spaces [85].
Computational Platform We use AMD EPYC 7232P CPU in the computation of *NASGraph*. To compute the performance of baselines requiring GPUs, a single NVIDIA A40 GPU is used.

S.1.3 Combining outputs from preceding graph blocks

When there are multiple graph blocks connected to the same graph block, *i.e.* graph blocks combine outputs from multiple preceding graph blocks, there are two ways to combine the outputs: *summation* and *concatenation*. In the case of summation, we do the forward propagation for each channel of all branches. In the case of concatenation, however, the outputs of the preceding graph blocks do not match the input dimension of the current graph block. We add virtual channels to the outputs of the preceding graph blocks such that the output dimension matches the input dimension, and hence we can do the conversion in the block-wise fashion. The virtual channels are all-zeros matrix $\mathbb{O}$. We want to emphasize that the summation or concatenation is determined by the original neural architecture. The graph block just combines components in the neural architecture, such as Conv and ReLU. *w.l.o.g.* we examine the case of concatenation and the case of summation of two preceding graph blocks connecting to the l-th graph block as shown in Figure S1. In the case of summation, two graph blocks connect to the l-th graph block. The output of each graph block has 4 channels. The input of l-th graph block has 4 channels. In the case of concatenation, two outputs from the preceding graph blocks have 2 channels, while the input of l-th graph block has 4 channels.
Summation The summation requires that outputs of the preceding graph blocks have the same dimension as the input of the current graph block. We perform forward propagation for each channel of the outputs of the preceding graph blocks. Hence, there are 8 forward propagations in the case shown in

Figure S1. Each forward propagation determines the scores between the "activated" channel and all the output channels of l-th graph block.

Concatenation The concatenation requires that outputs of the preceding graph blocks have the same dimension as the input of the current graph block except for the channel dimension, and the summation of the channel size of the outputs of the preceding graph blocks equal to the channel size of the input of the current graph block. Because we do the conversion block-wise, there will be a dimension mismatch if we do forward propagation using the dimension of the preceding outputs. Therefore, we added virtual channels to make sure the two dimensions match. The number of added channels is $C^{(l)} - C^{(l-1)}$. In the case of Figure S1, two virtual channels are added for each input. We do not "activate" virtual channels in any forward propagation, so there are 4 forward propagations in total.

S.1.4 Variation in Rankings in Different Runs

The training-free NAS methods do a single forward/backward propagation on models with randomly initialized parameters, it is potentially subjected to different random initialization. To examine the variation in rankings due to different initialization of model parameters, we repeat the computation of metrics 8 times. Each run initializes model parameters following Gaussian distribution. We use the pair rank difference to indicate the variation in the rankings for a pair of two random processes. The pair rank difference is defined by:

$$\text{pair rank difference} = \sum_{\mathrm{k=0}}^{\mathrm{n_a}} \left|\mathrm{rank_i(a_k)} - \mathrm{rank_j(a_k)}\right| , \tag{S1}$$

where n_a is the total number of neural architectures, on NAS-Bench-201 benchmark, $n_a = 15,625$. $\mathrm{rank_i(a_k)}$ and $\mathrm{rank_j(a_k)}$ are the ranking of k-th architecture in the i-th and j-th random initialization processes, respectively. Considering the computational overheads, we choose NASWOT, a NAS method that requires a training dataset, to compute the variation in rankings in comparison with our method. We use 8 random seeds and compute the pair rank difference. Figure S6 shows the pair rank difference on NAS-Bench-201 and different datasets. Since our method is data-agnostic, it has the same mean and standard deviation across different datasets. Our metric has a contiguously smaller variation in the rankings of neural architectures. We believe one of the reasons is that the NASWOT method, or other data-dependent NAS methods, has a random selection of minibatches and the random initialization of model parameters. Our method, on the other hand, is not subjected to the random selection of minibatches by using fixed inputs. When we compute ranking correlations between the performance of neural architectures and graph properties, we find negligible differences among 8 random initializations. So, we believe that our method is not subjected to random initialization.

S.1.5 Top-K Retrieval Performance

The ranking correlation comparison exhibits the advantage of the proposed method. In addition to the ranking correlation, we examine the performance comparison using top-k retrieval performance. In the top-k retrieval calculation, we extract architectures with top $k\%$ predicted performance and then examine the fraction of architectures that are in the set of real top $k\%$ best architectures. By varying k, we compute the area under the curve (AUC) as the metric. Figure S9 shows the performance comparison. The result is consistent with the comparison of ranking correlations.

S.1.6 Ablation Study

We examine the performance of surrogate models with a different number of channels and a different number of cells. There are two reducing factors: (1) the number of channels c and (2) the number of search cells e within one module. The random search results on the NAS-Bench-201 benchmark are listed in Supplementary Materials Table S9 for the case $N = 100$ and $N = 200$. GT reports the highest test accuracy of neural architectures within the selected subset in the random search process. In both

$N = 100$ and $N = 200$ cases, we do not find a significant variation in the performance of surrogate models except for the efficiency. As we decrease the number of cells or channels, there is a significant improvement in efficiency. We use a grid routine to systematically study the effect of the number of channels and number of cells on the performance of the surrogate model. Figure S8 shows the effect of varying numbers of channels and several cells within the same module on NAS-Bench-201 using a random search algorithm. Each search process is repeated for 100 times.
Number of channels Along x axis in Figure S8 shows the effect of varying the number of channels. When the number of channels is 16, the surrogate model performs best. When the number of channels is 1, there is a pronounced degradation in the performance.
Number of cells Reducing the number of channels is a common practice for one-shot NAS to reduce the size of the supernet. We examine this routine in the *NASGraph* framework. Along y axis in Figure S8 shows the effect of changing the number of cells within a module. We find there is no monotonous increase nor decrease in the performance as the number of cells varies. We believe it is related to the way we bridge neural architecture space and graph space. By using synthetic data, the input to each graph block is independent. Besides, the cell structure is the same within the same module. Considering the cell structure corresponding to a subgraph, the module consisting of a linear stack of cell structures corresponds to a stack of identical subgraphs. Therefore, subgraph properties using surrogate models that decrease the number of cells are expected to impose an insignificant effect on the graph properties and, hence, rankings of the original neural architectures.
Overall, we find no remarkable difference in the accuracy using the random search algorithm. However, we can vastly boost efficiency by using surrogate models. We choose the surrogate model *NASGraph*(16, 1, 3) to compute graph properties on different NAS benchmarks.
In addition to the random search, we examine the ranking correlation between the model performance and graph properties for different surrogate models. We choose the surrogate model *NASGraph*(4, 5, 3) and *NASGraph*(16, 1, 3). Four graph properties are computed using these two surrogate models: `density`, `avg_deg`, `resilience`, and `wedge`. The definition of these four graph properties is listed in Supplementary Materials Table S8. Supplementary Materials Table S10 shows the performance on the NAS-Bench-201 benchmark, and Supplementary Materials Table S11 shows the performance on the TransNAS-Bench-101 benchmark. We do not observe a significant difference by using different surrogate models.

Input initialization We use all-ones matrices as the synthetic inputs to get an unbiased estimation of the contribution of different graph blocks to the output. Table S12, Table S13 and Table S13 show the performance comparison when the synthetic input is randomly initialized: $x_{ij}^{(\ell)} \sim \mathcal{N}(0, 1)$. Results indicate that randomly initialized inputs cannot correctly build the relationship between inputs and outputs for graph blocks.

Threshold value We use a threshold value to determine whether to build a graph edge. Table S12, Table S13 and Table S13 show the performance comparison when using the threshold value of 1. A performance degradation is observed.

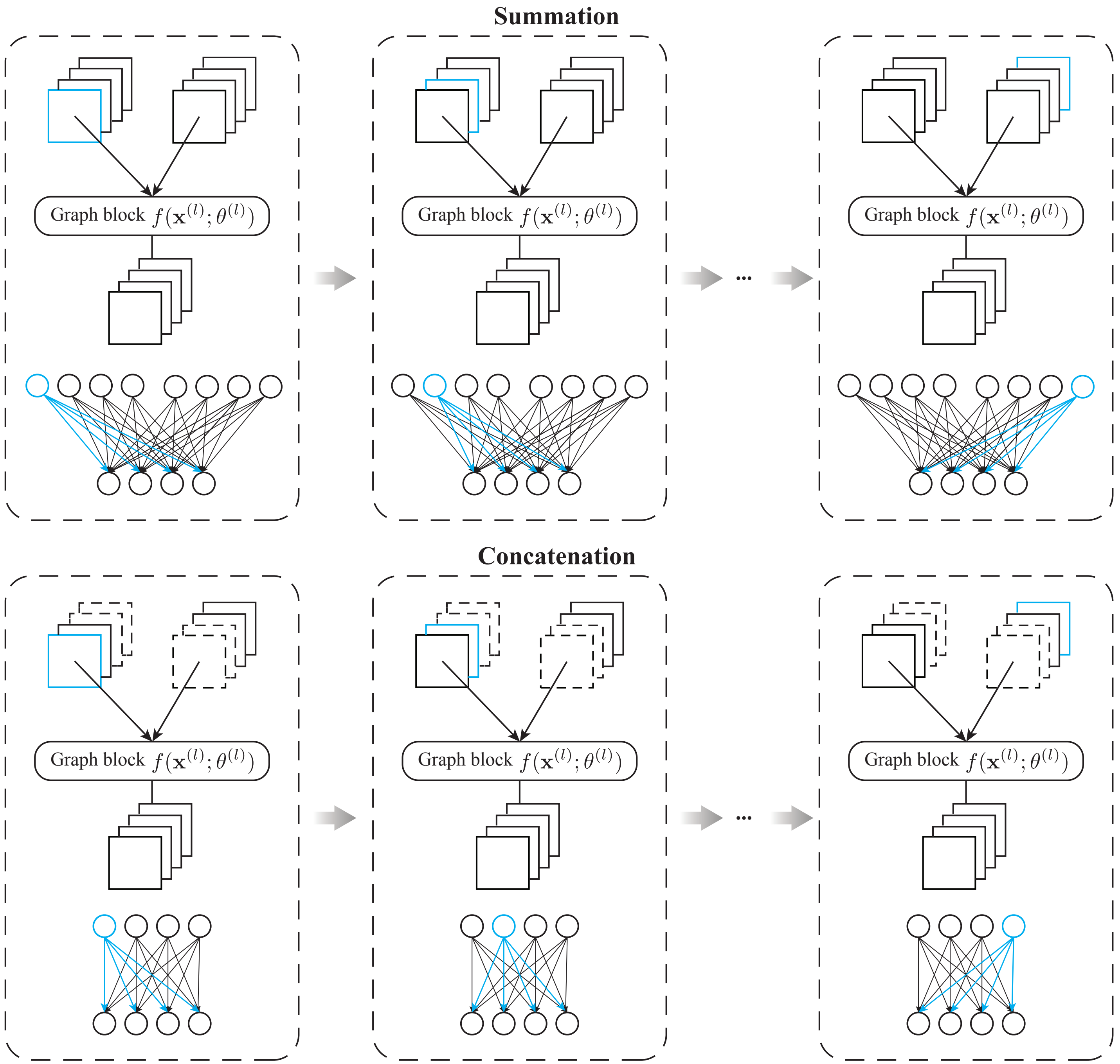

Figure S1: An illustration of converting one graph block of the neural architecture to a subgraph in the case of summation and concatenation. Channels in dashed lines mean virtually concatenated channels. These channels become a zero matrix after applying a mask. Connection is established if the score between two graph nodes is non-zero.

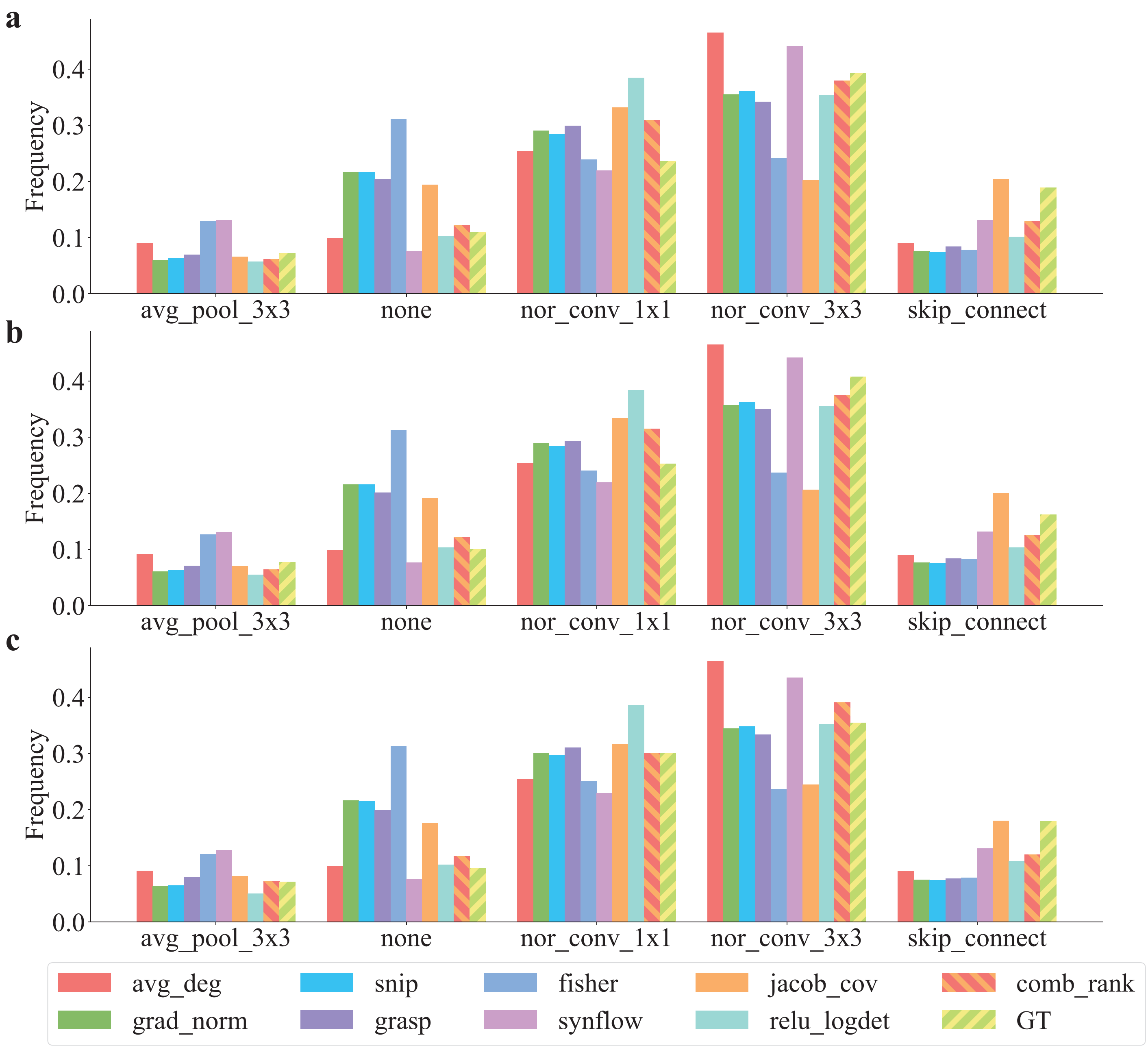

Figure S2: The preference for different operations for the NAS-Bench-201 benchmark for different datasets. (a) CIFAR-10. (b) CIRFAR-100. (c) ImageNet-16-120. GT is the operation distribution of top architectures ranked by test accuracy. The frequency distribution difference compared to GT indicates the bias of NAS methods towards operations. The combination of `avg_deg` and `jacob_cov` has the lowest bias.

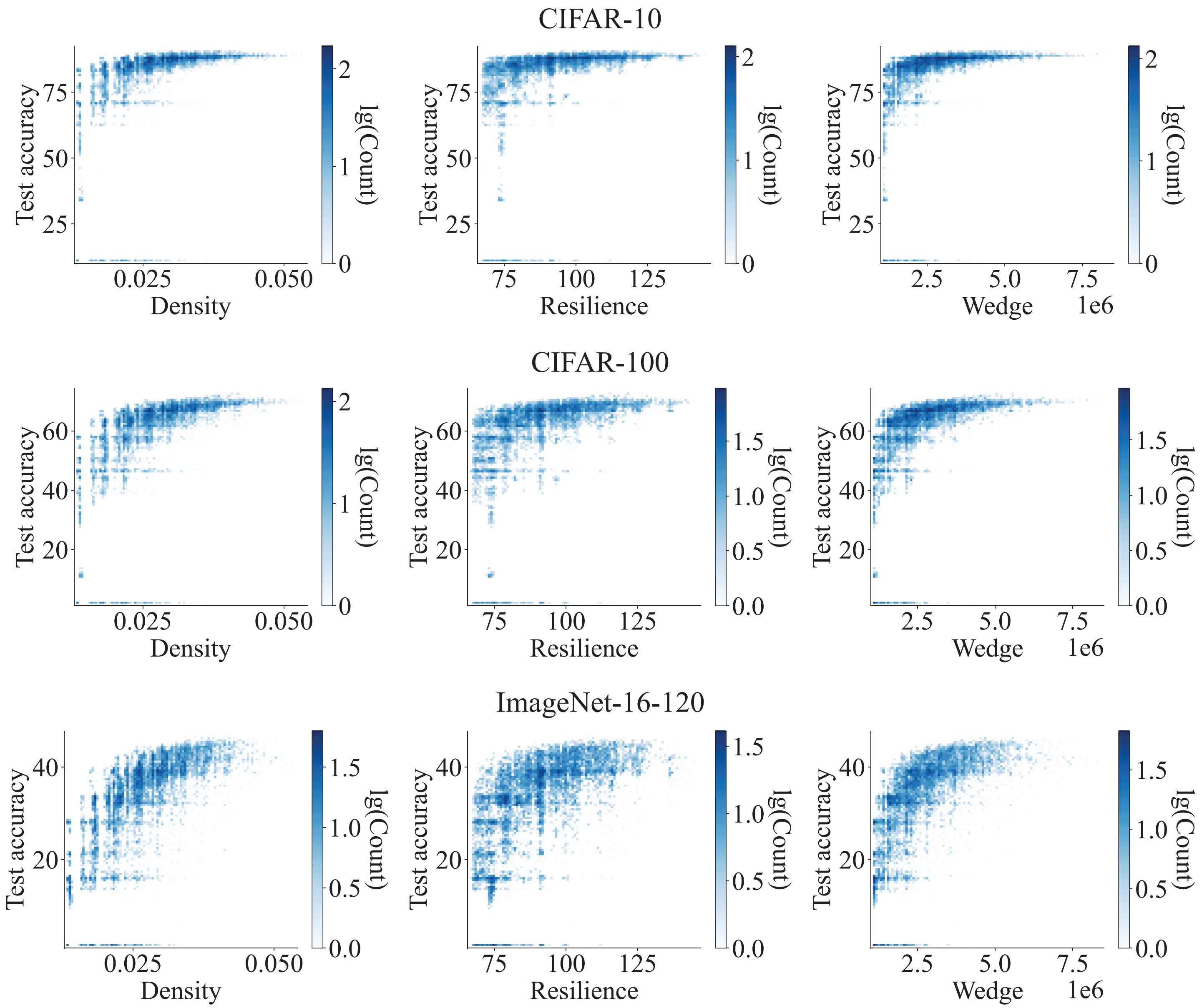

Figure S3: The ranking correlations between properties of the converted graphs and test accuracy of the corresponding neural architectures on the NAS-Bench-201 benchmark. 3 graph properties (*NASGraph* metrics) are examined: density (`density`), resilience parameter (`resilience`) and wedge count (`wedge`).

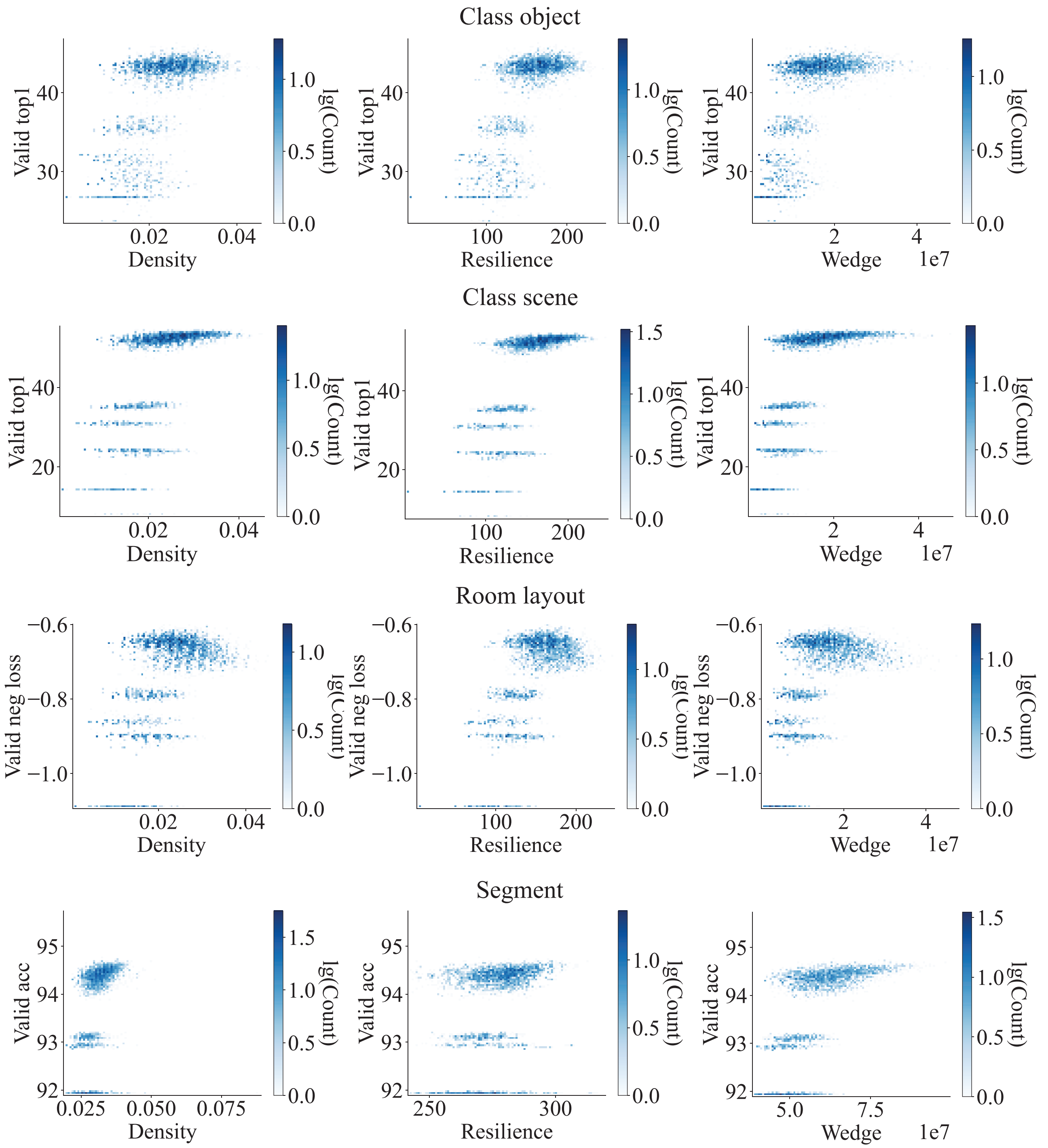

Figure S4: The ranking correlations between properties of the converted graphs and test accuracy of the corresponding neural architectures on the TransNAS-Bench-101 benchmark. Three graph properties (*NASGraph* metrics) are examined: density (`density`), resilience parameter (`resilience`) and wedge count (`wedge`). There are outliers (neural architectures with considerably poorer performance) that are not shown in the visualization due to the scale limit.

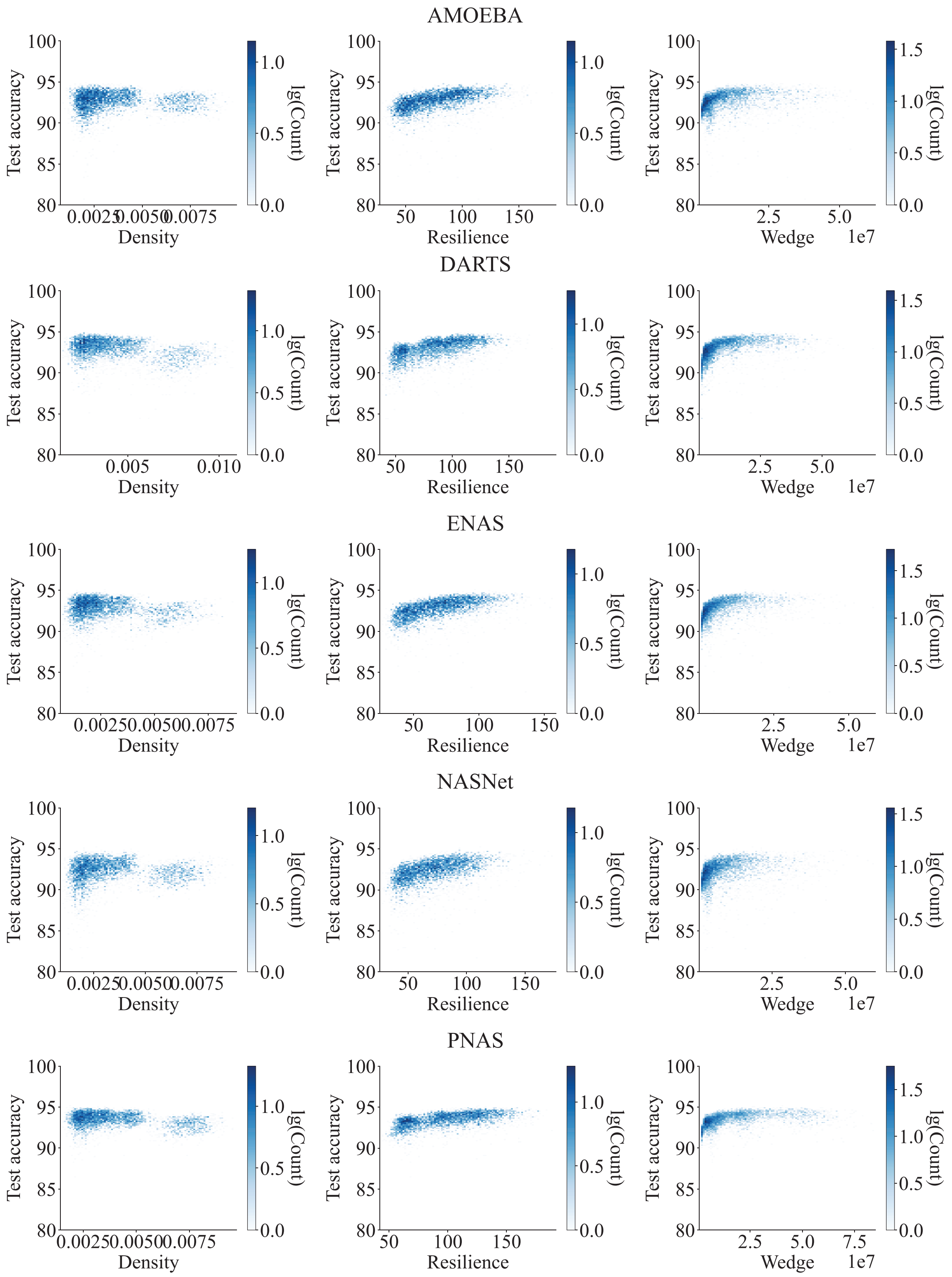

Figure S5: The ranking correlations between properties of the converted graphs and test accuracy of the corresponding neural architectures on the NDS benchmark. Three graph properties (*NASGraph* metrics) are examined: density (`density`), resilience parameter (`resilience`) and wedge count (`wedge`).

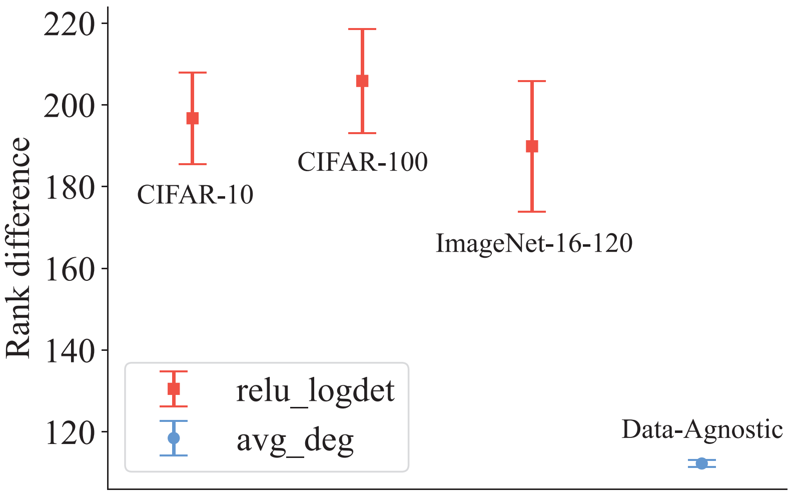

Figure S6: The variation of the architecture rankings using `relu_logdet` and `avg_deg` as the ranking metric. Architectures in the NAS-Bench-201 benchmark are ranked using training-free NAS metrics. The mean value and standard deviation are calculated over 8 random seeds.

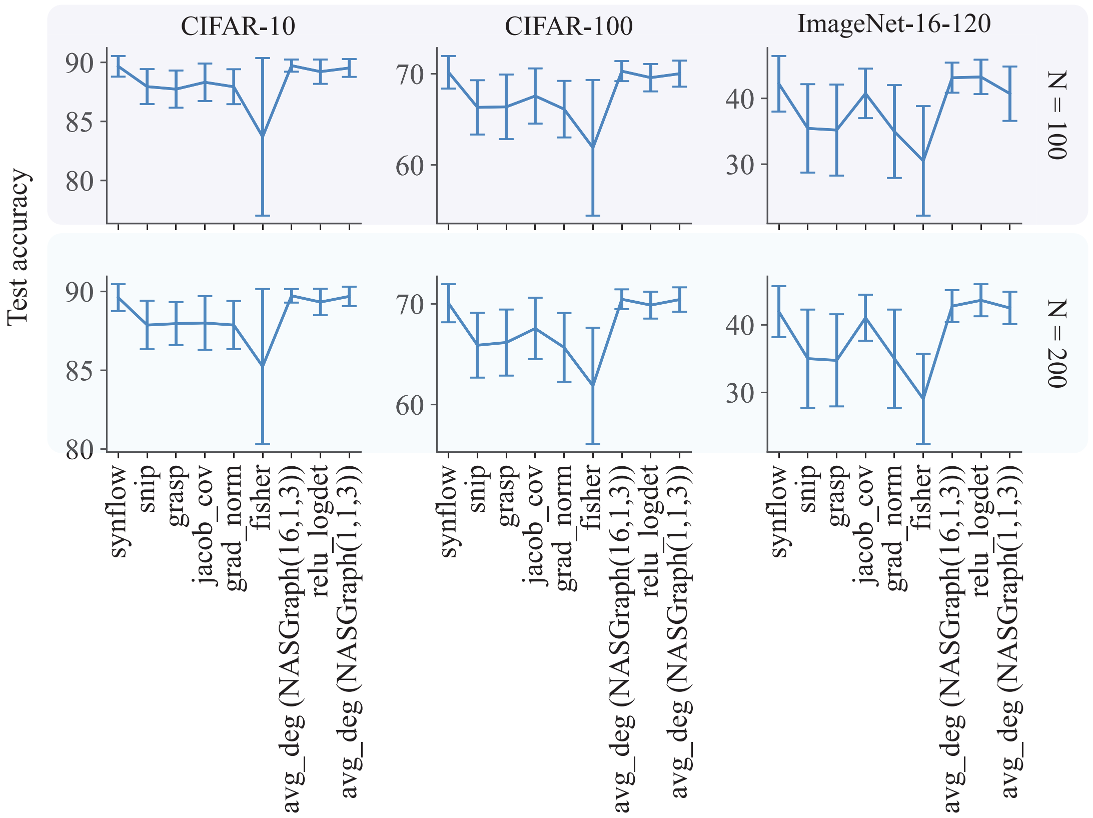

Figure S7: Comparison of test accuracies using the random search. Architectures in NAS-Bench-201 are searched and their performance on CIFAR-10, CIFAR-100 and ImageNet-16-120 is extracted to examine the search effectiveness. The *NASGraph* method achieves the performance comparable to the best baseline method while the computational cost is remarkably reduced.

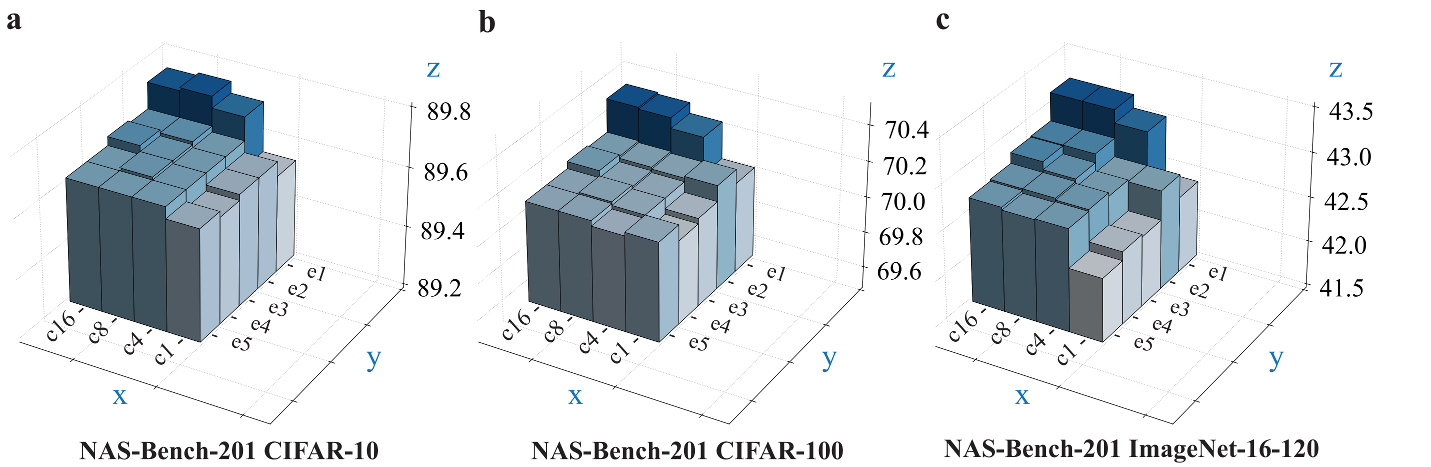

Figure S8: 3D-Bar chart for the reducing factors. The number of channels c and number of cells e within a module change in the ablation study. We examine 4 different number of channels along the x axis: $c \in \{1, 4, 8, 10\}$ and 5 different number of cells along the y axis: $e \in \{1, 2, 3, 4, 5\}$. The z axis is the test accuracies of neural architectures.

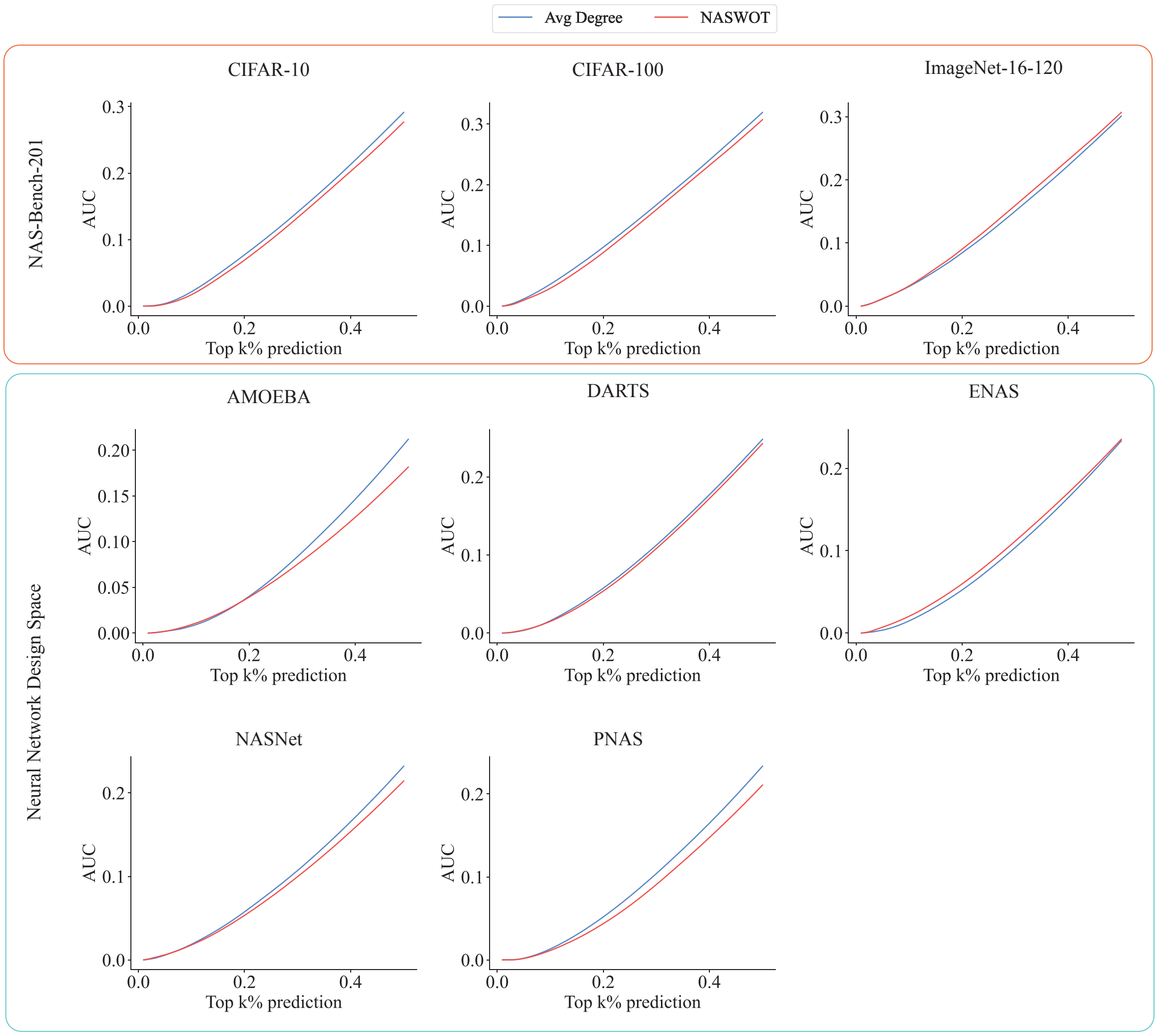

Figure S9: Comparison of AUC for the top-k retrieval performance curve on the NAS-Bench-201 benchmark and the NDS benchmark.

Table S1: Comparison of the ranking correlation between *NASGraph* and training-free NAS methods using single metric on NAS-Bench-201. Correlations are calculated between the metrics and test accuracies. ↑ indicates that the larger magnitude the correlation has, the better metric is.

Method	Metric	CIFAR-10		CIFAR-100		ImageNet-16-120	
		$\rho\uparrow$	$\tau\uparrow$	$\rho\uparrow$	$\tau\uparrow$	$\rho\uparrow$	$\tau\uparrow$
NASWOT	`naswot`	0.76	0.57	0.79	**0.61**	0.71	0.55
ZiCo†	`zico`	0.74	0.55	0.78	0.58	0.76	0.56
TENAS	NTK	-	-	-	-0.42	-	-
	NLR	- -		- -0.50		- -	-
Zero-Cost NAS	`grad_norm`	0.58	0.42	0.64	0.47	0.58	0.43
	`snip`	0.58	0.43	0.63	0.47	0.58	0.43
	`grasp`	0.48	0.33	0.54	0.38	0.56	0.40
	`fisher`	0.36	0.26	0.39	0.28	0.33	0.25
	`synflow`	0.74	0.54	0.76	0.57	0.75	0.56
	`jacob_cov`	0.73	0.55	0.71	0.55	0.71	0.54
Ours	`avg_deg`	**0.78**	**0.58**	**0.80**	0.60	**0.77**	**0.57**

† Original implementation of ZiCo uses cutout data augmentation [86]. To make a fair comparison, we recalculate the correlation without cutout data augmentation.

Table S2: Comparison of Kendall's Tau ranking correlations τ between test accuracies and training-free NAS metrics on the NDS benchmark. The benchmark consists of five NAS search spaces: AMOEBA, DARTS, ENAS, NASNet and PNAS. $\tau\uparrow$ indicates that the larger magnitude τ has, the higher the ranking correlation is.

Metric	$\tau\uparrow$				
	AMOEBA	DARTS	ENAS	NASNet	PNAS
`grad_norm`	-0.12	0.22	0.06	-0.05	0.15
`snip`	-0.09	0.26	0.10	-0.02	0.18
`grasp`	0.02	-0.04	0.03	0.18	-0.01
`fisher`	-0.12	0.19	0.04	-0.07	0.14
`jacob_cov`	0.22	0.19	0.11	0.05	0.10
`synflow`	-0.06	0.30	0.14	0.04	0.21
`zico`	-0.01	0.36	0.21	0.10	0.20
`naswot`	0.22	**0.47**	0.37	0.30	0.38
`avg_deg` (Ours)	**0.32**	0.45	**0.41**	**0.37**	**0.40**

Table S3: Comparison of Spearman's ranking correlations ρ between validation accuracies and training-free NAS metrics on micro TransNAS-Bench-101. The baseline performance is extracted from [66]. Note that `synflow` and `avg_deg` are data-agnostic. CO is `class_object`, CS is `class_scene`, RL is `room_layout`, SS is `segment_semantic`. ρ $\uparrow$ indicates that the larger magnitude ρ has, the higher the ranking correlation is.

Metric	$\rho\uparrow$			
	CO	CS	RL	SS
`plain`	0.34	0.24	0.36	-0.02
`grasp`	-0.22	-0.27	-0.29	0.00
`fisher`	0.44	0.66	0.30	0.12
`epe_nas`	0.39	0.51	**0.40**	0.00
`grad_norm`	0.39	0.65	0.25	0.60
`snip`	0.45	0.70	0.32	0.68
`synflow`	0.48	0.72	0.30	0.00
`l2_norm`	0.32	0.53	0.18	0.48
`params`	0.45	0.64	0.30	0.68
`zen`	0.54	0.72	0.38	0.67
`jacob_cov`	0.51	**0.75**	**0.40**	**0.80**
`flops`	0.46	0.65	0.30	0.69
`naswot`	0.39	0.60	0.25	0.53
`zico`	0.55	0.68	0.26	0.61
Our method				
`avg_deg`	**0.55**	0.70	0.37	0.66
Optimal graph property†	**0.62**	**0.75**	**0.47**	0.68

† The optimal graph property extracts the best correlation among 4 graph properties: `avg_deg`, `density`, `resilience` and `wedge`.

Table S4: Comparison of the accumulated frequency difference between training-free NAS methods and GT on top 10% architectures of the NAS-Bench-201. GT ranks architectures ranked by test accuracies. Lower value means less bias (*i.e.* closer to GT).

Metric	Bias $\downarrow$			
	CIFAR-10	CIFAR-100	ImageNet-16-120	Average
`relu_logdet`	0.3	0.27	0.19	0.25
`grad_norm`	0.32	0.3	0.24	0.29
`snip`	0.31	0.29	0.24	0.28
`grasp`	0.31	0.28	0.24	0.28
`fisher`	0.52	0.52	0.53	0.52
`synflow`	0.22	0.18	0.27	0.22
`jacob_cov`	0.39	0.42	0.22	0.34
Our method				
`avg_deg`	0.22	**0.14**	0.27	0.21
`comb_rank`	**0.17**	0.17	**0.12**	**0.15**

Table S5: Comparison of the ranking correlations using multiple metrics with training-free NAS methods on NAS-Bench-201. Correlations between the combined metric and NAS Benchmark performance are reported. TENAS [69] combines rankings by NTK and NLR. Zero-Cost NAS [40] takes a majority vote among the three metrics: `synflow`, `jacob_cov` and `snip`. Our method combines the rankings of `avg_deg` and `jacob_cov`.

Method		CIFAR-10		CIFAR-100		ImageNet-16-120	
		$\rho\uparrow$	$\tau\uparrow$	$\rho\uparrow$	$\tau\uparrow$	$\rho\uparrow$	$\tau\uparrow$
TENAS	Rank combine	-	-	-	0.64	-	-
Zero-Cost NAS	Voting	0.82	-	0.83	-	0.82	-
Ours	Rank combine	**0.85**	0.66	**0.85**	**0.67**	**0.82**	0.64

Table S6: Comparison of the performance of the searched optimal architecture on Imagenet under the mobile setting. † denotes the performance obtained in our experiments. Otherwise, performance is extracted from [69].

Method	Params (M)	Test Accuracy	Search Method
NASNet-A	5.3	74.0	RL
AmoebaNet-C	6.4	75.7	evolution
PNAS	5.1	74.2	SMBO
MnasNet-92	4.4	74.8	RL
DRATS (2nd)	4.7	73.3	gradient
SNAS (mild)	4.3	72.7	gradient
GDAS	5.3	74.0	gradient
BayesNAS	3.9	73.5	gradient
P-DARTS (CIFAR-10)	4.9	75.6	gradient
P-DARTS (CIFAR-100)	5.1	75.3	gradient
PC-DARTS (CIFAR-10)	5.3	74.9	gradient
PC-DARTS (ImageNet)	5.3	75.8	gradient
ProxylessNAS (GPU)	7.1	75.1	gradient
TE-NAS (CIFAR-10)	6.3	73.8	training-free
TE-NAS (ImageNet)	5.4	75.5	training-free
ZiCo†	5.5	75.0	training-free
NASGraph† (*avg_ deg*)	5.6	75.7	training-free

Table S7: Comparison of training-free NAS metrics using the random search method. The same subset of architectures is randomly chosen from NAS-Bench-201 for all metrics. GT reports the performance of the best architecture in that subset. The wall-clock time comparison reveals the high efficiency of the *NASGraph* method.

Metric	Running time ↓	CIFAR-10		CIFAR-100		ImageNet-16-120	
		Valid ↑	Test ↑	Valid ↑	Test ↑	Valid ↑	Test ↑
N = 100							
`relu_logdet`	52.72 GPU sec.	89.51 ± 0.96	89.22 ± 1.03	69.48 ± 1.44	69.58 ± 1.50	42.92 ± 2.41	43.27 ± 2.62
`grad_norm`	364.68 GPU sec.	88.28 ± 1.42	87.94 ± 1.48	65.96 ± 3.11	66.13 ± 3.10	34.97 ± 6.82	34.96 ± 7.06
`snip`	363.71 GPU sec.	88.29 ± 1.42	87.95 ± 1.48	66.14 ± 2.96	66.32 ± 2.97	35.44 ± 6.49	35.44 ± 6.72
`grasp`	377.29 GPU sec.	88.06 ± 1.55	87.74 ± 1.58	66.27 ± 3.50	66.38 ± 3.55	35.20 ± 6.76	35.19 ± 6.93
`fisher`	315.57 GPU sec.	84.08 ± 6.68	83.70 ± 6.67	61.77 ± 7.26	61.89 ± 7.44	30.80 ± 8.02	30.49 ± 8.33
`synflow`	360.15 GPU sec.	89.91 ± 0.87	89.67 ± 0.88	70.03 ± 1.79	70.17 ± 1.79	41.89 ± 4.13	42.23 ± 4.24
`jacob_cov`	360.48 GPU sec.	88.68 ± 1.56	88.32 ± 1.59	67.45 ± 2.91	67.57 ± 3.03	40.64 ± 3.54	40.76 ± 3.77
`avg_deg` (NASGraph(1, 1, 3))	7.78 CPU sec.	89.74 ± 0.77	89.53 ± 0.75	69.90 ± 1.38	70.01 ± 1.43	42.00 ± 2.80	40.73 ± 4.14
`avg_deg` (NASGraph(16, 1, 3))	106.18 CPU sec.	89.95 ± 0.49	89.73 ± 0.52	70.17 ± 1.06	70.29 ± 1.10	42.72 ± 2.33	43.15 ± 2.29
GT	-	90.98 ± 0.36	90.77 ± 0.31	71.48 ± 0.86	71.69 ± 0.81	45.45 ± 0.67	45.74 ± 0.65
N = 200							
`relu_logdet`	90.39 GPU sec.	89.64 ± 0.81	89.33 ± 0.84	69.65 ± 1.36	69.87 ± 1.33	43.25 ± 2.22	43.62 ± 2.37
`grad_norm`	644.23 GPU sec.	88.23 ± 1.51	87.87 ± 1.53	65.46 ± 3.34	65.67 ± 3.42	35.08 ± 7.05	35.00 ± 7.26
`snip`	712.58 GPU sec.	88.23 ± 1.51	87.87 ± 1.54	65.68 ± 3.16	65.89 ± 3.21	35.08 ± 7.05	35.00 ± 7.26
`grasp`	692.74 GPU sec.	88.31 ± 1.35	87.96 ± 1.37	65.97 ± 3.21	66.16 ± 3.28	34.83 ± 6.63	34.74 ± 6.81
`fisher`	622.92 GPU sec.	85.55 ± 4.91	85.24 ± 4.92	61.69 ± 5.62	61.86 ± 5.77	29.39 ± 6.38	29.04 ± 6.65
`synflow`	742.74 GPU sec.	89.87 ± 0.85	89.61 ± 0.85	69.93 ± 1.84	70.05 ± 1.89	41.54 ± 3.76	41.93 ± 3.77
`jacob_cov`	688.77 GPU sec.	88.34 ± 1.67	88.00 ± 1.71	67.39 ± 2.93	67.55 ± 3.05	40.95 ± 3.24	41.04 ± 3.41
`avg_deg` (NASGraph(1, 1, 3))	15.98 CPU sec.	89.92 ± 0.61	89.69 ± 0.62	70.25 ± 1.20	70.42 ± 1.21	41.96 ± 2.44	42.48 ± 2.39
`avg_deg` (NASGraph(16, 1, 3))	217.21 CPU sec.	89.96 ± 0.38	89.73 ± 0.43	70.22 ± 0.99	70.45 ± 0.98	42.27 ± 2.36	42.76 ± 2.36
GT	-	91.14 ± 0.25	90.91 ± 0.24	71.84 ± 0.76	72.04 ± 0.72	45.72 ± 0.54	46.01 ± 0.50

Table S8: Definition and computation complexity of the four graph properties used in *NASGraph*. The performance of using average degree is reported in the main manuscript while that of other properties is reported in the Supplementary Materials.

	Average degree	Density	Resilience parameter [79]	Wedge count [80]
Definition	$\bar{k} = \frac{1}{n_g} \sum_{i \in V} k_i$	$d_G = \frac{m_g}{(n_g - 1)}$	$\beta_{\text{eff}} = \frac{\mathbf{1}^T \mathbf{A} \mathbf{s}^{\text{in}}}{\mathbf{1}^T \mathbf{A} \mathbf{1}}$	$\mathcal{W}_G = \sum_{i \in V} \binom{k_i}{2}$
Time complexity	$\mathcal{O}(m_g + n_g)$	$\mathcal{O}(m_g + n_g)$	$\mathcal{O}(n_g^2 + m_g)$	$\mathcal{O}(m_g + n_g)$

Table S9: Comparison of different surrogate models on NAS-Bench-201. `avg_deg` is used as the metric to score architectures. Reported results are averaged over 100 runs, and both mean values and standard deviations of accuracies associated with the highest scores are recorded. GT records the highest accuracies of the randomly sampled architectures. The time reported is the CPU running time.

Method	Time ↓	CIFAR-10		CIFAR-100		ImageNet-16-120	
		Valid ↑	Test ↑	Valid ↑	Test ↑	Valid ↑	Test ↑
N = 100							
NASGraph(1, 1, 3)	7.78 sec.	89.74 ± 0.77	89.53 ± 0.75	69.90 ± 1.38	70.01 ± 1.43	42.00 ± 2.80	40.73 ± 4.14
NASGraph(4, 1, 3)	19.23 sec.	89.91 ± 0.49	89.70 ± 0.52	70.05 ± 1.16	70.22 ± 1.18	42.60 ± 2.43	43.00 ± 2.42
NASGraph(8, 1, 3)	62.61 sec.	89.96 ± 0.46	89.74 ± 0.49	70.15 ± 1.01	70.28 ± 1.05	42.72 ± 2.33	43.15 ± 2.30
NASGraph(16, 1, 3)	106.18 sec.	89.95 ± 0.49	89.73 ± 0.52	70.17 ± 1.06	70.29 ± 1.10	42.72 ± 2.33	43.15 ± 2.29
NASGraph(1, 5, 3)	25.63 sec.	89.78 ± 0.64	89.58 ± 0.65	69.93 ± 1.23	70.06 ± 1.30	41.80 ± 2.66	42.22 ± 2.63
NASGraph(4, 5, 3)	89.68 sec.	89.84 ± 0.53	89.62 ± 0.55	69.88 ± 1.07	70.04 ± 1.13	42.22 ± 2.56	42.66 ± 2.56
NASGraph(8, 5, 3)	258.03 sec.	89.84 ± 0.52	89.62 ± 0.55	69.90 ± 1.07	70.07 ± 1.14	42.18 ± 2.57	42.65 ± 2.57
GT	-	90.98 ± 0.36	90.77 ± 0.31	71.48 ± 0.86	71.69 ± 0.81	45.45 ± 0.67	45.74 ± 0.65
N = 200							
NASGraph(1, 1, 3)	15.98 sec.	89.92 ± 0.61	89.69 ± 0.62	70.25 ± 1.20	70.42 ± 1.21	41.96 ± 2.44	42.48 ± 2.39
NASGraph(4, 1, 3)	61.60 sec.	89.95 ± 0.38	89.73 ± 0.42	70.17 ± 1.01	70.42 ± 0.99	42.25 ± 2.33	42.73 ± 2.32
NASGraph(8, 1, 3)	186.21 sec.	89.97 ± 0.37	89.74 ± 0.42	70.25 ± 0.89	70.47 ± 0.87	42.40 ± 2.11	42.87 ± 2.13
NASGraph(16, 1, 3)	217.21 sec.	89.96 ± 0.38	89.73 ± 0.43	70.22 ± 0.99	70.45 ± 0.98	42.27 ± 2.36	42.76 ± 2.36
NASGraph(1, 5, 3)	50.33 sec.	89.80 ± 0.59	89.61 ± 0.59	70.05 ± 1.15	70.18 ± 1.23	41.47 ± 2.50	41.97 ± 2.51
NASGraph(4, 5, 3)	196.08 sec.	89.89 ± 0.45	89.68 ± 0.49	70.05 ± 0.93	70.21 ± 1.05	41.91 ± 2.04	42.45 ± 2.05
NASGraph(8, 5, 3)	413.47 sec.	89.88 ± 0.45	89.66 ± 0.49	70.05 ± 0.95	70.23 ± 1.07	42.01 ± 2.07	42.53 ± 2.09
GT	-	91.14 ± 0.25	90.91 ± 0.24	71.84 ± 0.76	72.04 ± 0.72	45.72 ± 0.54	46.01 ± 0.50

Table S10: Comparison between the surrogate models *NASGraph*(16, 1, 3) and *NASGraph*(4, 5, 3). Three datasets are used: CIFAR-10, CIFAR-100, ImageNet-16-120. Four graph properties are used: `density`, `avg_deg`, `resilience` and `wedge`

Methods	Metric	NAS-Bench-201					
		CIFAR-10		CIFAR-100		ImageNet-16-120	
		$\rho \uparrow$	$\tau \uparrow$	$\rho \uparrow$	$\tau \uparrow$	$\rho \uparrow$	$\tau \uparrow$
NASGraph(4, 5, 3)	`density`	0.75	0.56	0.77	0.57	0.75	0.55
	`avg_deg`	0.76	0.57	0.78	0.58	0.76	0.56
	`resilience`	0.74	0.54	0.75	0.55	0.73	0.53
	`wedge`	0.76	0.57	0.78	0.58	0.76	0.56
NASGraph(16, 1, 3)	`density`	0.77	0.57	0.78	0.59	0.75	0.56
	`avg_deg`	0.78	0.58	0.80	0.60	0.77	0.57
	`resilience`	0.68	0.49	0.69	0.50	0.67	0.48
	`wedge`	0.77	0.57	0.79	0.59	0.76	0.56
Optimal single metric		0.78	0.58	0.80	0.60	0.77	0.57

Table S11: Ranking correlations ρ and τ between the validation accuracies and *NASGraph* metrics using different surrogate models. The structure of modules and cells is encoded in the arch string such as `64-41414-1_02_333` (we refer the reader to [65] for details on the arch string). 4 channels and 16 channels refer to c in *NASGraph*(c, n, m).

Metric	Micro TransNAS-Bench-101							
	CO		CS		RL		SS	
	$\rho\uparrow$	$\tau\uparrow$	$\rho\uparrow$	$\tau\uparrow$	$\rho\uparrow$	$\tau\uparrow$	$\rho\uparrow$	$\tau\uparrow$
4 Channels								
`avg_deg`	0.56	0.39	0.71	0.51	0.38	0.25	0.67	0.48
`density`	0.54	0.37	0.68	0.49	0.37	0.24	0.60	0.43
`resilience`	0.62	0.44	0.75	0.55	0.47	0.31	0.33	0.23
`wedge`	0.60	0.42	0.74	0.54	0.43	0.28	0.68	0.49
16 Channels								
`avg_deg`	0.55	0.38	0.70	0.50	0.37	0.24	0.66	0.47
`density`	0.53	0.36	0.68	0.48	0.35	0.22	0.55	0.39
`resilience`	0.62	0.43	0.74	0.54	0.47	0.31	0.34	0.24
`wedge`	0.59	0.41	0.74	0.54	0.42	0.27	0.68	0.49
Optimal Single Metric	0.62	0.44	0.75	0.55	0.47	0.31	0.68	0.49

Table S12: Ablation study on using different input initialization and different threshold values on the NASBench201 benchmark. `avg_deg` is used as the metric to rank neural architectures and ranking correlations are computed.

Method	CIFAR-10		CIFAR-100		ImageNet-16-120	
	$\rho\uparrow$	$\tau\uparrow$	$\rho\uparrow$	$\tau\uparrow$	$\rho\uparrow$	$\tau\uparrow$
All-ones matrix as input	0.78	0.58	0.80	0.60	0.77	0.57
Randomly initialized matrix as input	-0.01	0.00	-0.01	0.00	0.00	0.00
Different threshold	0.77	0.57	0.79	0.60	0.76	0.57

Table S13: Ablation study on using different input initialization and different threshold values on the NDS benchmark. `avg_deg` is used as the metric to rank neural architectures and Kendall's Tau ranking correlation is computed.

Metric	$\tau\uparrow$				
	AMOEBA	DARTS	ENAS	NASNet	PNAS
All-ones matrix as input	0.32	0.45	0.41	0.37	0.40
Randomly initialized matrix as input	0.00	0.01	-0.01	0.00	0.00
Different threshold	0.23	0.30	0.28	0.25	0.28

Table S14: Ablation study on using different input initialization and different threshold values on the TransNASBench101 benchmark. `avg_deg` is used as the metric to rank neural architectures and Spearman's ranking correlation is computed.

Method	$\rho\uparrow$			
	CO	CS	RL	SS
All-ones matrix as input	0.55	0.70	0.37	0.66
Randomly initialized matrix as input	0.00	0.01	0.02	-0.02
Different threshold	0.23	0.47	0.18	0.32
